# Genome-wide association study and genetic diversity analysis on nitrogen use efficiency in a Central European winter wheat (*Triticum aestivum* L.) collection

**DOI:** 10.1371/journal.pone.0189265

**Published:** 2017-12-28

**Authors:** István Monostori, Fruzsina Szira, Alessandro Tondelli, Tamás Árendás, Krisztián Gierczik, Luigi Cattivelli, Gábor Galiba, Attila Vágújfalvi

**Affiliations:** 1 Agricultural Institute, Centre for Agricultural Research, Hungarian Academy of Sciences, Martonvásár, Hungary; 2 CREA Research Centre for Genomics and Bioinformatics, Fiorenzuola d’Arda (PC), Italy; 3 Festetics Doctoral School, Georgikon Faculty, University of Pannonia, Keszthely, Hungary; Institute of Genetics and Developmental Biology Chinese Academy of Sciences, CHINA

## Abstract

To satisfy future demands, the increase of wheat (*Triticum aestivum* L.) yield is inevitable. Simultaneously, maintaining high crop productivity and efficient use of nutrients, especially nitrogen use efficiency (NUE), are essential for sustainable agriculture. NUE and its components are inherently complex and highly influenced by environmental factors, nitrogen management practices and genotypic variation. Therefore, a better understanding of their genetic basis and regulation is fundamental. To investigate NUE-related traits and their genetic and environmental regulation, field trials were evaluated in a Central European wheat collection of 93 cultivars at two nitrogen input levels across three seasons. This elite germplasm collection was genotyped on DArTseq® genotypic platform to identify loci affecting N-related complex agronomic traits. To conduct robust genome-wide association mapping, the genetic diversity, population structure and linkage disequilibrium were examined. Population structure was investigated by various methods and two subpopulations were identified. Their separation is based on the breeding history of the cultivars, while analysis of linkage disequilibrium suggested that selective pressures had acted on genomic regions bearing loci with remarkable agronomic importance. Besides NUE, genetic basis for variation in agronomic traits indirectly affecting NUE and its components, moreover genetic loci underlying response to nitrogen fertilisation were also determined. Altogether, 183 marker-trait associations (MTA) were identified spreading over almost the entire genome. We found that most of the MTAs were environmental-dependent. The present study identified several associated markers in those genomic regions where previous reports had found genes or quantitative trait loci influencing the same traits, while most of the MTAs revealed new genomic regions. Our data provides an overview of the allele composition of bread wheat varieties anchored to DArTseq® markers, which will facilitate the understanding of the genetic basis of NUE and agronomically important traits.

## Introduction

Wheat is grown on greater land area than any other commercial crops; its production has exceeded 700 million tons in the recent years, hence wheat became the most important food grain source for humans [1]. Considering the predicted population growth, the increase in per capita consumption and the changes in diets, the global production of agricultural products needs to be higher by 60 percent by 2050. So we face the challenge to increase the average wheat yield from the current 3.4 t/ha to 5.42 t/ha. In addition, cereal production already utilises more than half of the global fertiliser production [2]. As a consequence, fertiliser consumption is increasing: Growth in the amount of applied nitrogen (N) fertiliser is expected to double by 2050 from 112 Mt (2015) to 236 Mt. [3–4]. Nevertheless, the utilisation of N fertilisers is rather inefficient. Approximately 50-70% of the applied N vanishes from the plant-soil system enriching the reactive N compounds in the atmosphere, polluting the ground and surface waters. The environmental damages associated with the utilisation of N-based fertilisers are becoming significant not just at local but also regional and global scales [5–6]. The effect of the negative environmental and economic impacts could be reduced through better agronomic practices and the utilisation of N-efficient cultivars with improved N use efficiency (NUE) [7]. The world market demands high-quality bread wheat with high protein content and strong gluten properties. However, with the improvement of NUE, as an adverse effect, the grain protein content (GPC) may decrease. The inverse relationship between grain yield (GY) and GPC makes it difficult to improve these two traits simultaneously. Accordingly, it is a challenging task to reduce the excessive input of N fertilisers while maintaining the desired yield and GPC.

NUE in agricultural systems is defined as GY per unit of available N (from the soil and/or fertiliser) [8], which can be influenced by numerous traits (reviewed by [9]). The most immediate goal is to reach better NUE by the improvement of N uptake efficiency (NUpE)–the ability of the plant to remove N from the soil–and to improve N harvest index (NHI)–the remobilisation of the stored N into the grain [10–11]. It is also known that different limiting factors are involved in plant metabolism for maximising NUE under high or low N availability [12].

Moreover, the importance of the two NUE components, NUpE and N utilisation efficiency (NUtE) are variable, mostly depending on the amount of the available N. Previous studies revealed that NUpE contributes more to genetic variation in NUE, especially at low N levels, whereas at high N levels, variation in NUE is mainly due to differences in NUtE (reviewed by) [11–13]. Consequently, when N availability is not a limiting factor, NUtE can be more dominant. Twenty winter wheat varieties were studied [14] at two N levels and it was found that NUpE accounted for more of the variation in NUE at low N condition (64%) than at high N supply (30%). More importantly, 63% of the genotype × N level interaction was explained for NUpE. In contrast, an investigation studying 39 winter wheat cultivars at five N levels found that cultivar differences in NUpE occurred only at the three highest N supply levels and it was concluded that NUtE explained more of the variation in GY than NUpE, regardless of the level of soil N [15]. These experiments prove that NUE and its components are highly influenced by environmental factors: the interaction of climate- nutrient- water availability, N management and genotypic variation [12]. Therefore, it is important to understand the interactions between NUE-related traits as well as their genetic and environmental regulation.

Since NUE is determined by multiple genetic factors and influenced heavily by the environment, its genetic dissection is quite challenging [12]. In addition, the large genotype by environment interaction makes the investigation even more difficult. It was concluded that the discovery of a major gene (with big effect size) controlling NUE itself, as it is, is quite unlikely. Rather, the description of many genes or QTLs (Quantitative Trait Loci) with minor or moderate effect will take us closer to the understanding of the final picture, the complexity of NUE [13]. These minor QTLs are more likely are specific QTLs representing genetic variation specific for the species or variety and the environmental conditions [13]. To discover many of these specific QTLs it is inevitable to investigate NUE in different environments studying diverse genetic material.

Numerous studies have established that significant genetic variation exists for NUE-related traits in wheat. Components of NUE were investigated in bread wheat under five N levels, and depending on N the supply, a 24-42% difference was fund in NUtE between the elite wheat varieties [15]. Another study revealed significant differences (69–88%) for N remobilisation efficiency and N translocation efficiency (90-93%) among five wheat genotypes [16]. These results indicate that genetic potential indeed exists to improve NUE in wheat.

Genetic diversity is a fundamental aspect of crop improvement; therefore, the effective utilisation of genetic resources in breeding programs is essentialy as long as this diversity integrates positive and profitable genes [17]. I has been concluded [13] that QTL data obtained from wide crosses, in spite of the fact that they have generated relatively robust QTL information dataset, may not be relevant in developing modern cereal crops. Diverse elite genetic materials, which are well-adapted and commercially relevant for specific interests are more useful to identify allelic variation for superior NUE. In this way, knowledge gained from scientific studies can provide valuable results in plant breeding.

Many genes or QTLs influencing NUE have been successfully mapped in wheat under various N supplies. Most of the studies utilised bi-parental populations to identify the genetic basis of NUE and its associated traits [7, 18–20].

Association mapping exploits ancestral recombination events that occurred throughout the evolutionary history of the populations and takes into account all the segregating alleles present in the population. In contrast, due to the restricted number of meiotic events, the genetic resolution of the segregation based conventional bi-parental maps often remain insufficient. Moreover, only the alleles from the parental genotypes are scrutinised in these ‘classical’ bi-parental analyses. So, compared to the capabilities of bi-parental mapping association analysis is a more efficient approach to dissect complex quantitative traits [21]. Concerning that this method is relatively new in cereal genetics, only a few genome-wide association studies (GWAS) have been published in wheat for NUE on diverse genetic materials under various different conditions so far [17, 22–25].

In order to identify chromosomal regions involved in the determination of NUE and its related traits, a genome-wide association study was carried out in a European winter wheat collection under different conditions. The Marker-Trait-Associations (MTA) detected in commercially relevant genetic materials will facilitate the isolation of agronomically important genes, especially those related to NUE.

## Materials and methods

### Plant materials and experimental design

A set of 93 bread wheat (*Triticum aestivum* L.) varieties (S1 Table) were phenotyped in Martonvásár, Hungary, at the MTA ATK (Centre for Agricultural Research, Hungarian Academy of Sciences), in six environments (3 year × 2 N levels), during three successive cropping seasons (2012-2013, 2013-2014 and 2014-2015). The examined cultivars represent an elite germplasm collection grown mainly in Hungary and in Central Europe; however, some old (e.g. ‘Bezostaja-1’, ‘Bánkúti 1201’) or non-continental (e.g. ‘Nudakota’) varieties were also involved. Cultivars were selected according to their involvement and importance in the Hungarian wheat breeding.

In three consecutive years adjacent fields belonging to the Agricultural Institute of MTA ATK (47°18ʹ N, 18°48ʹ E, 105 MASL) were used. Each cultivar was sown between 2 and 21 October, in a split-plot design, in three replicates, at two N levels: (1) no nitrogen supply–considered as extensive management (referred as N0) and (2) intensive management, whereby 120 kg N per hectare (referred as N120) was applied. In the N0 treatment, only the naturally occurring nitrogen was available in the soil, while in the case of N120, N was top-dressed at growth stage (GS) 21-24 [26]. In 2014 and 2015 the fertiliser treatment was applied on 7 and 17 March, respectively. In 2013, the spring was cold and wet, so the N fertiliser could be allocated to the field only on 17 April (at tillering stage too, GS 21–26). In 2013, ammonium nitrate (34% N), while in 2014 and 2015, calcium ammonium nitrate (27% N) was allocated. The size of each plot was 3×1.44 m consisting of 12 rows, containing 500 viable seeds/m^2^. Sowing date and seed rate were applied according to the Hungarian practice [27–28]. N treatment was considered as main plots and varieties as sub-plots. P_2_O_5_ and K_2_O fertilisation and plant protection were applied according to standard agricultural practice and no growth regulators were used. Crops were combine-harvested at state of grain maturity in the period of 8-21 July. Each spring, soil samples were collected before fertilisation from two depths (0-0.3 m and 0.3-0.6 m) and mineral N (ammonium + nitrate) contents (N_mineral_) and main soil properties were determined in accredited laboratory according to NAT-1-1093/2001 (NÉBIH NTAI, Velence, Hungary). Soil type at each location was chernozem, but they differed in their naturally available N content. The average N_mineral_ content (mean value of six soil samples in each year) was 21, 494 and 78 kgha^-1^ in 2013, 2014 and 2015, respectively. Weather data (i.e. daily precipitation and mean temperature values) were also recorded in the Martonvásár region. Further information about soil parameters and meteorological data can be obtained from [29].

### Phenotypic evaluation

The entire collection was evaluated for 16 agronomically important or N input-related traits at two N input levels (N0 and N120). The investigated traits were heading date (HD), plant height (PH), GY, thousand grain weight (TGW), weight of straw–referred to as straw yield (SY), spike number per meter (SN), grain number per spike (GN), harvest index (HI), N harvest index (NHI), GPC, NUE, NUpE, NUtE, amount of N taken up by the whole plant (NUp_full_), amount of the N harvested in grain (NUp_grain_), grain N accumulation efficiency (GNACE). GY, HD and PH were scored on a per plot basis, while GN, SN, TGW and SY were evaluated from above-ground biomass samples collected from a representative row of each plot, whose length was one meter. HD was assigned when 50% of the spikes within a given plot had fully emerged (GS 59) [26] and expressed in days from sowing, while PH was measured in cm at maturity. The one meter samples were dried for 48 h at 70°C and SY was determined and transformed in kgha^-1^. SN and GN were determined on per meter basis. GN was determined using Contador seed counter machine (Pfeuffer GmbH, Kitzingen, Germany) and TGW was also calculated. The number of fully developed spikes was calculated from the one meter sample from each plot and expressed in spikes per m. GY was assigned when plots were combine-harvested at plant maturity and calculated in kg hectare^-1^ as SY. HI was determined as the ratio of GY and above-ground biomass (both of them expressed in kgha^-1^), which is the sum of GY and SY. The straw and grain samples were milled and N content was determined by Dumas method [30] using Rapid NIII nitrogen analyser (Elementar Analysensysteme GmbH., Hanau, Germany). GPC in w/w% was calculated as grain N concentration multiplied by a coefficient of 5.8. The amount of the N harvested in grain and in straw was calculated by multiplying the N concentration obtained from Rapid NIII analysis with the amount of GY and SY, respectively, and expressed in kg hectare^-1^. NUE, NUtE and NUpE were defined according to [8] as follows: NUE is the GY divided by the sum of naturally available N content in the soil and the amount of N allocated with fertiliser in kg hecatare^-1^ (N_soil_). NUtE is the GY divided by NUp_full_. NUp_full_ is the sum of NUp_grain_ and N harvested in straw, which was expressed in kg hectare^-1^. NUpE is the NUp_full_ divided by N_soil_. NHI is the ratio of NUp_grain_ to NUp_full_, which is a measure of N translocation efficiency. GNACE is NUp_grain_ divided by the N_soil_, which serves as a measure of the overall efficiency.

Analysis of variance for all traits in each of the three consecutive years was performed using General Linear Model (GLM) procedure with SPSS 22.0 software for Windows. Correlations between phenotypic traits were also calculated through SPSS 22.0 in each cropping year and treatment.

### Genotyping and marker selection

Genomic DNA was extracted by the Qiagen DNeasy Plant Mini Kit according to the manufacturer’s instructions and sent to the commercial service provider of DArT marker, Triticarte Pty. Ltd. (Canberra, Australia; http://diversityarray.com/). Genotyping was performed with the wheat DArTseq® platform and generated two types of marker data: (1) genotyping by sequencing (GBS) markers are SNP markers obtained by sequencing the fragments derived from genome complexity reduction and subsequent SNP calling, and (2) silicoDArT markers referring to the presence or absence of restriction fragment in the genomic representation. SilicoDArT is analogous to microarray DArT but extracted *in silico* from the sequences obtained from the genomic representation used for GBS genotyping. Genomic sequences of fragments from both types of markers were also available. A detailed description of the platform used to genotype the collection can be found in [31]. As a result of genotyping, the initial dataset contained 12,293 polymorphic codominant SNP and 13,160 dominant silicoDArT markers. This marker set was filtered on the basis of individual marker-related statistics, provided by the Triticarte Pty. Ltd. The minimum threshold value for call-rate and reproducibility was 95%. Since it was assumed that all genotypes are homozygous, DArTseq® markers showing heterozygous calls were indicated as missing and markers with >5% heterozygous alleles were excluded. Additionally, markers with minor allele frequency lower than 0.1 were also removed from the analysis. After filtering marker data, a total of 4,201 polymorphic DArTseq® markers (2,700 silicoDArTs and 1,501 SNPs) were obtained and used for population structure analysis. However, since only 3,290 of these polymorphic markers had been previously mapped (data obtained from Triticarte Pty. Ltd), the unmapped markers were not included in linkage disequilibrium (LD) and marker trait associations analysis. A reduced marker set consisting of 300 unlinked markers were also used to complement and verify the population structure analysis. They were obtained from the filtered set by excluding markers, which were localised closer than 5 cM to any other marker.

### Analysis of population structure

To determine the underlying population structure in the Hungarian wheat collection, different methodologies were used and compared. Firstly, the Bayesian algorithm implemented in the software package Structure. v2.3.4 [32] was used to estimate the number of hypothetical subpopulations (K) and to assign cultivars to them. Structure runs were performed with two marker set (4,201 markers and a reduced set with 300 markers) applying the admixture model with correlated allele frequencies. A burn-in of 100,000 iteration followed by 100,000 Markov Chain Monte Carlo iterations were set for accurate parameter estimates. The value of K was evaluated from 1 to 8, with 5 iterations for each K value. The most likely number for subpopulations was estimated by following the ΔK method, described by [33]. Each cultivar was assigned to one subpopulation based on the membership probability. Principal Coordinate Analysis (PCoA) was also used as an alternative way of visualizing the genetic stratification within the collection, by means of the software package PAST v.3.12 [34]. Additionally, the phylogenetic relationship among the cultivars was estimated using PAST software using the neighbour joining method. Bootstrap values for 1,000 replicates are indicated. Analysis of molecular variance (AMOVA) and genome wide estimation of population differentiation using Wright’s F-statistics (F_ST_) among subpopulations were performed with software package Arlequin 3.5.2.2 [35] to investigate levels of genetic variation revealed by Structure analysis.

### Linkage disequilibrium analysis

Genome-wide LD analysis was performed using the LD function in the software TASSEL 5.0 [36]. Intra-chromosomal LD was estimated for the entire population. The analysis comprised the pairwise estimated squared allele-frequency correlation (r^2^), normalized coefficient of linkage disequilibrium (D-prime or D’) and the significance of each pair of loci. The locus were considered to be in significant LD when p<0.05. To estimate the LD decay, significant r^2^-values were plotted against the genetic distance between the marker-pairs and a second-degree smoothed Loess curve was fitted using SPSS 22.0. [37]. The interception of the Loess curve and background LD (critical r^2^) was considered as an estimate of LD decay. The critical r^2^-value was determined by root transforming the unlinked r^2^-values and taking the 95^th^ percentile of the distribution as the threshold beyond which LD is likely caused by genetic linkage [23,38]. According to [39], marker pairs with a distance above 50 cM were considered as unlinked. LD decay was estimated separately in all chromosomes, for the three genomes (A, B and D) in the entire population.

### Marker-trait association analysis

Genome-wide association analysis was performed using TASSEL 5.0 software [36] for each measured and calculated trait and for genotype response to N fertilisation too. Response to N fertilisation (RN) was estimated as the ratio between N120 and N0 for GY, SN, GN, GPC, NHI, NUPfull, NUpgrain and NUtE. In the association analysis each cultivar was represented as the phenotypic mean of the three plots in each season and the seasons were analysed separately. Four different statistical models were adopted to calculate P-values for MTAs to avoid spurious associations: (1) general linear model (GLM) with Q-matrix as correction for population structure; (2) GLM with Principal component analysis (PCA) as correction for population structure; (3) mixed linear model (MLM) with Kinship-matrix (K-matrix) as correction for population structure and (4) MLM with Q-matrix and K-matrix as correction for population structure [32;40]. PCA and K-matrix were calculated with TASSEL 5.0 considering recommendations of [41]. MLM was run without compression and selecting the option “population parameters previously determined”. The critical threshold for assessing the significance of MTAs were calculated by false discovery rate separately for each trait in each year [42]. An MTA was defined significant if the calculated q-value passed the FDR threshold for a given trait in all of the four models. When a trait from the same environment (i.e. year and treatment) was found to be associated with more than one marker, that MTA was considered, which possessed its highest effect size within a 5 cM region.

Moreover quantile–quantile plots of -log_10_ P-values in S3 Fig illustrate the relationship between the observed and the expected dataset based on the MLM+K+Q model. Appropriate control for population structure and relatedness can be seen, since empirical distribution do not deviate from expectations.

## Results

### Genotypic data

Genotyping the Hungarian wheat collection with DArTseq® platform resulted in a final dataset comprising 4,201 quality-filtered, polymorphic DArTseq® markers of which 3,290 were placed on the genetic map. Among the mapped markers, 1,631 were located on the B genome, 1,210 on the A genome and only 449 on the D genome. Their chromosomal distribution can be found in S1 Fig. The total map length covered 5,880 cM. The average distance between markers was 1.79 cM, 1.22 cM and 5.77 cM in the A, B and D genome, respectively. Neither A nor B genome had gaps larger than 50 cM. However, the largest gap on chromosomes 3D, 4D and 5D were 60.42 cM, 74.21 cM and 64.55 cM, respectively. In summary, D genome presented the largest and the highest number of gaps among the genomes.

### Analysis of population structure

**Fig 1:**
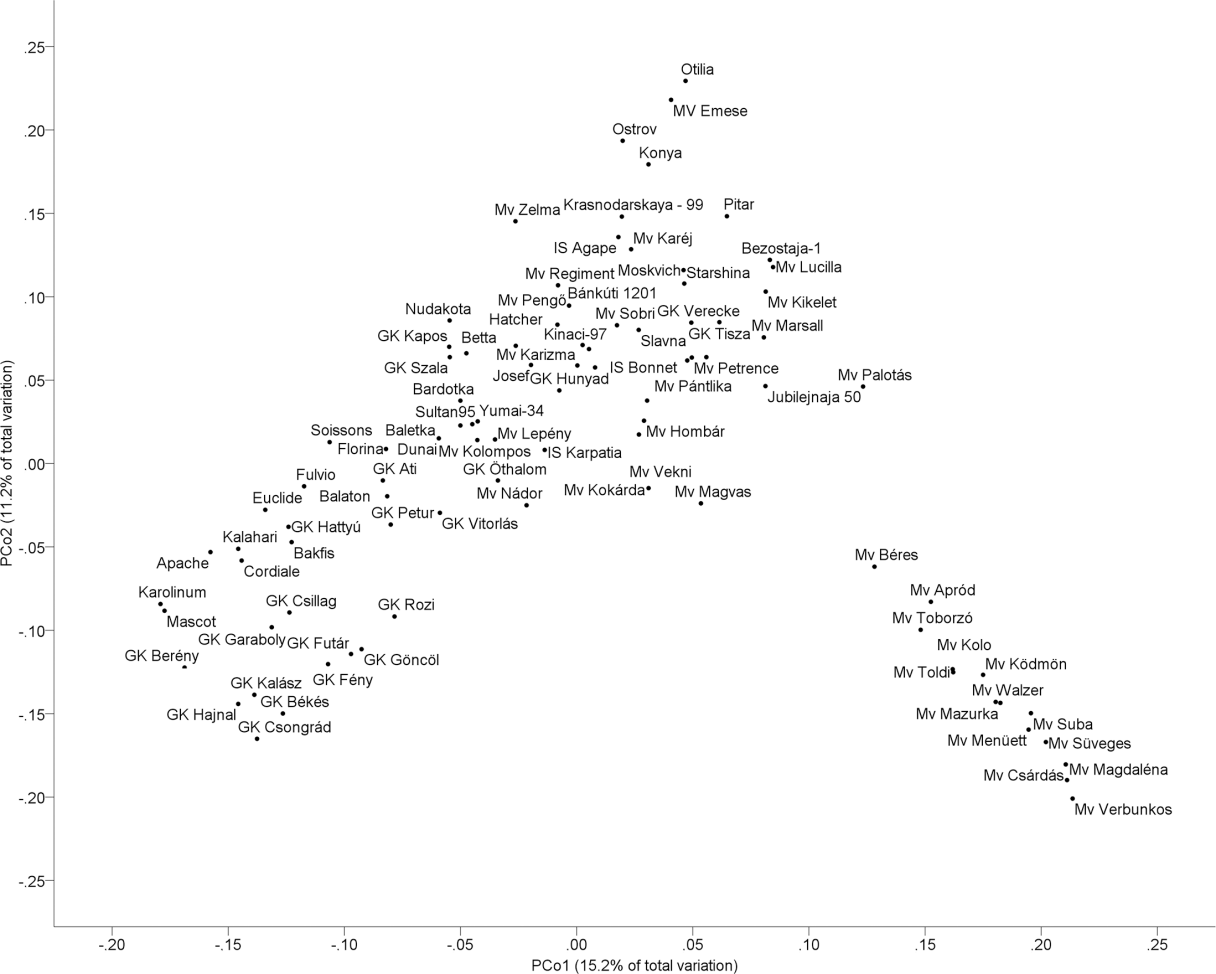
Principal coordinate analysis of 93 winter wheat genotypes based on Jaccard similarity index. PCo 1 and PCo 2 are the first and second principal coordinates, respectively, and the numbers in parentheses refer to the proportion of variance explained by the principal coordinates.

To analyse the genetic diversity within the collection, the relatedness of the cultivars was firstly investigated with the Bayesian approach implemented in Structure software [32]. Evidence of significant population structure was provided by the analysis based on the method of [33]. The maximum ΔK value occurred at K = 2, so two subpopulations (referred as Sp1 and Sp2) were identified. The Q matrix (membership probability estimates) was extracted from Structure runs and each cultivar was assigned to one of the two subpopulations based on a membership probability >0.51. The Sp1 contains the majority of the cultivars (79 cultivars), while Sp2 consisted of the remaining 14 cultivars. The separation of the two subpopulations reflects the breeding history of the cultivars. Since the algorithms in the Structure software assume independent loci, measured on randomly sampled unrelated individuals, another Bayesian clustering was carried out with a reduced marker set. The analysis of these 300 unlinked markers also led to a very similar result, two subpopulations were identified. PCoA was used as an alternative way of analysing and visualising population structure. The first two principal coordinates explained 15.2% and 11.2% of the molecular variance, and the separation into two subpopulations was confirmed by this independent analysis (Fig 1). The DArTseq® marker-based phylogenetic structure of the wheat collection with the two subpopulations, Sp1 and Sp2 is presented in S2 Fig.

The distribution of molecular variation among and within the two subpopulations was estimated by AMOVA, which revealed 23% (p≤0.001) of total genetic variation partitioned among subpopulations, whereas 77% of the variation was maintained within subpopulations. Additionally, the subpopulations were compared on the basis of the phenotypic data collected and significant differences were found in five out of six (3 year × 2 N level) environments with respect to GPC, NUpE, NUp_grain_ and NUp_full_, and in 4 cases with respect to GNACE, HI, GN, HD, NUE, GY (S2 Table).

### Linkage disequilibrium

**Fig 2:**
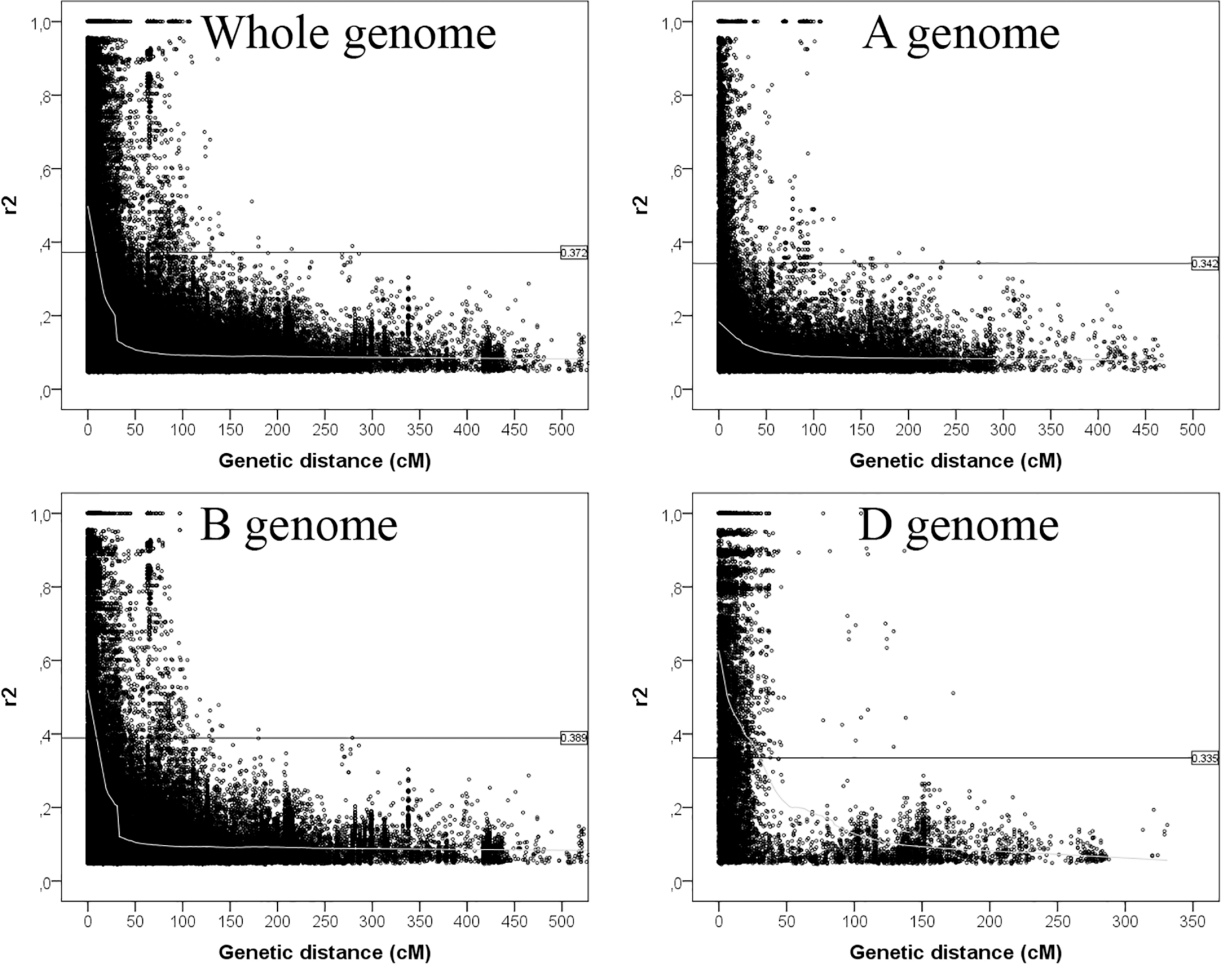
Intra-chromosomal LD (r^2^) decay of marker pairs in the whole genome and in the three wheat genomes as a function of genetic distance (cM). Horizontal line indicates the 95% percentile of the distribution of unlinked r^2^, which gives the critical r^2^-value. Second-degree LOESS fitting curve illustrates the LD decay (grey line).

LD was calculated for the entire population by pairwise marker r^2^ for each chromosome. The number of intra-chromosomal pairs, the number of significant marker pairs, critical r^2^, mean r^2^-values and the distributions of LDs are detailed in Table 1. Furthermore, LD analysis revealed differences between chromosomes (S3 Table). In the entire population 118,398 (35.2%) intra-chromosomal pairs showed a significant level of LD (Table 1). Analysis of the LD in the different wheat genomes revealed, that the highest number of the significant pairs and also the pairs that are in perfect LD (i.e., where r^2^ = 1 and D’ = 1) was found in B genome. Only 10,543 pairs showed significant LD in the D genome (compared to 29,405 in the A and 78,450 in the B genome) because of the lower marker density. On the other hand, the rates of significant and linked marker pairs were much higher on the D genome than on the A or B genome. The comparison of the mean r^2^-values showed that the D genome had a higher mean r^2^-value (0.221) with respect to the B (0.102) and A (0.062) genome. The highest mean r^2^-values occurred on 2D and 1B chromosomes, while the lowest ones on 3A, 7A, 3B and 7B chromosomes (S3 Table). The critical r^2^-value was quite similar in the three genomes, ranging from 0.2137 to 0.4371, with the maximum and minimum values on chromosomes 1B and 4D, respectively. The LD decay in the three genomes, including the Loess curve, is illustrated on Fig 2. The LD decay, estimated as the intercept of the Loess curve at the line of critical r^2^-value, was 9 cM for the whole genome and 9 cM and 30.5 cM for the B and D genomes, respectively. In the A genome the Loess curve did not intercept the critical line. The marker pairs in total LD had a mean distance of 8.44 cM in the entire population.

**Table 1:** Overview of intra-chromosomal LD in the whole genome and in the three genomes in the entire population.

	Marker pairs total	No. and rate^a^ of significant (p<0.05) marker pairs (%)	Mean r^2^ of all marker pairs	critical r^2^	No and rate^b^ of linked marker pairs (%)	No of marker pairs in perfect LD
A genome	106,384	29,405 (27.6%)	0.062	0.342	3,604 (12.2%)	971
B genome	209,186	78,450 (37.5%)	0.102	0.389	14,476 (18.5%)	3,785
D genome	21,010	10,543 (50.2%)	0.221	0.335	5,295 (50,2%)	855
Population	336,580	118,398 (35.2%)	0.097	0.372	23,084 (19.5%)	5,611

linked pairs: r^2^>critical r^2^, perfect LD: r^2^ = 1 and D’ = 1

^a^ the percentage of total marker pairs which were significant (p<0.05)

^b^ the percentage of significant marker pairs which had greater r^2^ than the critical r^2^

### Characterisation of the phenotypic traits

*Phenotypic variation*: The phenotypic performance of the entire population and the subpopulations across the six environments for the 15 investigated traits are shown in S2A Table. Large phenotypic differences were observed in the population for all traits. Grain yield ranged from 2.33 t ha^-1^ (in 2012-2013 at N0) to 6.65 t ha^-1^ (in 2013-2014 at N120). Strong environmental effect was observed, causing remarkable variation of phenotypic traits between seasons. For most of the traits greater differences have been found between seasons than between N treatments. However, N fertilisation had significant effect on most of the studied traits (S2A Table). The significant difference of TGW, NUtE, NHI and HI varied between seasons, while N fertilisation did not have significant effect on HD. The largest differences between N0 and N120 were detected for GY, NUp_grain_, and NUp_full_ (S2A Table). Other traits (except HD) also showed significant but moderate changes in response to N level. For example, HI changed only by 10%, while NUp_grain_ changed by 36% from the 3 years’ average. The values of NUE, NUpE and GNACE greatly varied between seasons, probably because these traits strongly depend on the theoretically available N in the soil, which was 21, 494 and 78 kgha^-1^ in three successive cropping seasons (2012-2013, 2013-2014 and 2014-2015). These results indicate that the environment (including the effect of different N_mineral_) had greater influence on the phenotypic variability than the 120 kgha^-1^ N fertiliser applied in each year. The diversity of NUE in each environment are shown in S4 Fig, which indicate that genetic potential indeed exists to improve NUE in wheat in all environment. Investigation of yield components revealed that N fertilisation increased SN and GN in all cropping seasons, while TGW decreased in the season of 2013-2014 due to N fertilisation. Additionally, the lowest GPC values were measured in 2013 and population means was almost the same in 2014 and 2015. Significant differences between the two subpopulations were observed for all of the traits (except PH) in at least one environment (S2B Table), but none of the traits showed significant difference in all environment. Consequently, the phenotypic difference between the two subpopulations was highly influenced by environmental effects. In the last season (2014–2015), when the plant nutrient availability was moderate, Sp2 significantly differed from Sp1 for most of the investigated traits.

*Analysis of variance components*: The ANOVA results of the different traits are presented in S4 Table. The highest proportion of the variance was explained by environmental effect for most of the traits except for TGW and HI. In their cases the genotypic factor was more relevant. These results also indicated that the environment (including the effect of different N_mineral_) was the most important factor in explaining the overall phenotypic variance, however, the input level and the genotype effect also had significant role in it. The genotype effect explained the second highest proportion of the phenotypic variance for most of the traits (9 traits). Genotype effect was highly significant for 14 traits, but not for NUpE and GNACE. Separate analysis of the years revealed that genotype had no significant effect on NUpE and GNACE in 2012-2013, when the lowest level of N_mineral_ was observed. The input level effect was significant for all traits except for TGW and HD (S4 Table); moreover, it explained the second highest proportion of the phenotypic variance for NUE, GNACE, NUpE, NUp_full_ and NUp_grain_. Among the two-way interactions, genotype × environment interaction explained the highest proportion of the variance for most of the traits and it was significant for 11 traits. Additionally, very strong input level × environment interaction (at p>0.001 level) was found for 10 traits and moderate effect for NUtE and SY (at p>0.05), while no significant input level × environment interaction was found for NUp_grain_, PH, SN and NUp_full_ (S4 Table).

*Correlations between traits*: Correlation analysis was performed separately for all environments (three cropping seasons, two N fertilisation levels) for the most relevant traits, and several significant correlations were found at p<0.01 level (Table 2). NUE was calculated as GY divided by available N_soil_; therefore, the correlation analysis of NUE and GY with any other trait in the six environments separately shows the same result, hence only NUE was visualised in the analysis. Higher correlation coefficient was observed between NUE (GY) and NUpE than between NUE (GY) and NUtE in all environments. Additionally, GNACE correlated very strongly with both NUE (GY) and NUpE. GPC showed a negative correlation with NUE (GY) and NUtE, except in the season of 2012-2013 when GPC was not significant with NUE (GY). NHI correlated positively with NUE (GY) and negatively with GPC in most environments. The correlation between NUE (GY) and GN was stronger than the correlation between NUE (GY) and SN, except in the season of 2013-2014. TGW was not correlated with NUE (GY), but TGW showed slightly negative correlation with SN and GN in most of the environments.

**Table 2:** Significant (p< 0.01) phenotypic trait correlations with mean values for each genotype for season 2012-2013 (A); 2013-2014 (B); 2014-2015 (C).

**A**											
	**NUE**	**NUpE**	**NUtE**	**NHI**	**HD**	**SN**	**TGW**	**GN**	**PH**	**GPC**	**GNACE**
**NUE**		0.779	NS	0.274	NS	0.305	NS	0.686	NS	NS	0.812
**NUpE**	0.831		-0.424	NS	NS	NS	NS	0.537	0.319	0.548	0.953
**NUtE**	NS	-0.482		0.384	NS	NS	NS	NS	NS	-0.733	-0.276
**NHI**	NS	NS	NS		NS	NS	NS	0.412	NS	NS	NS
**HD**	0.292	NS	NS	NS		NS	NS	NS	NS	NS	NS
**SN**	0.389	0.34	NS	-0.352	NS		-0.304	-0.342	NS	NS	NS
**TGW**	NS	NS	NS	NS	NS	-0.292		-0.283	NS	NS	NS
**GN**	0.704	0.542	NS	NS	0.461	NS	NS		NS	NS	0.624
**PH**	NS	NS	NS	NS	NS	NS	NS	NS		NS	NS
**GPC**	NS	0.386	-0.931	0.282	-0.332	NS	NS	NS	NS		0.54
**GNACE**	0.834	0.981	-0.491	NS	NS	0.289	NS	0.563	NS	0.413	
**B**											
	**NUE**	**NUpE**	**NUtE**	**NHI**	**HD**	**SN**	**TGW**	**GN**	**PH**	**GPC**	**GNACE**
**NUE**		0.716	0.286	0.582	0.277	0.658	NS	0.665	NS	-0.278	0.85
**NUpE**	0.671		-0.37	NS	NS	0.501	NS	0.439	NS	0.414	0.931
**NUtE**	0.549	NS		0.618	0.294	NS	NS	NS	NS	-0.772	NS
**NHI**	0.611	NS	0.763		0.473	0.314	NS	0.546	NS	-0.478	0.425
**HD**	0.309	NS	0.381	0.48		NS	NS	0.404	0.305	NS	NS
**SN**	0.574	0.486	NS	NS	NS		-0.389	NS	NS	NS	0.553
**TGW**	NS	NS	NS	NS	-0.295	-0.462		NS	NS	NS	NS
**GN**	0.509	NS	0.5	0.557	0.621	NS	-0.3		NS	NS	0.574
**PH**	NS	NS	NS	NS	0.393	NS	NS	NS		0.387	NS
**GPC**	-0.442	0.305	-0.93	-0.525	NS	NS	NS	-0.36	NS		NS
**GNACE**	0.809	0.929	NS	0.362	NS	0.466	NS	0.36	NS	NS	
**C**											
	**NUE**	**NUpE**	**NUtE**	**NHI**	**HD**	**SN**	**TGW**	**GN**	**PH**	**GPC**	**GNACE**
**NUE**		0.898	0.401	NS	NS	0.575	NS	0.687	NS	-0.327	0.939
**NUpE**	0.897		NS	NS	NS	0.64	NS	0.486	NS	NS	0.948
**NUtE**	0.58	NS		0.659	NS	NS	NS	0.527	NS	-0.738	NS
**NHI**	0.569	0.28	0.774		NS	NS	NS	0.499	NS	NS	0.282
**HD**	NS	NS	NS	NS		NS	NS	-0.275	NS	NS	NS
**SN**	0.494	0.567	NS	NS	NS		-0.327	NS	0.316	NS	0.523
**TGW**	NS	NS	NS	NS	NS	NS		-0.369	NS	NS	NS
**GN**	0.695	0.51	0.622	0.527	NS	NS	NS		NS	NS	0.623
**PH**	NS	NS	NS	NS	NS	0.331	NS	NS		NS	0.255
**GPC**	-0.275	NS	-0.694	NS	NS	NS	NS	-0.378	NS		-0.02
**GNACE**	0.957	0.94	0.407	0.576	NS	0.527	NS	0.62	NS	NS	

Above the diagonals the correlation coefficients of the extensive (N0), while below, for the intensive (N120) fertiliser management can be found (N0: 0 kg N hectare^-1^, N120: 120 kg N hectare^-1^); NS: non-significant

### Genome-wide mapping of agronomic and N-related traits

**Fig 3:**
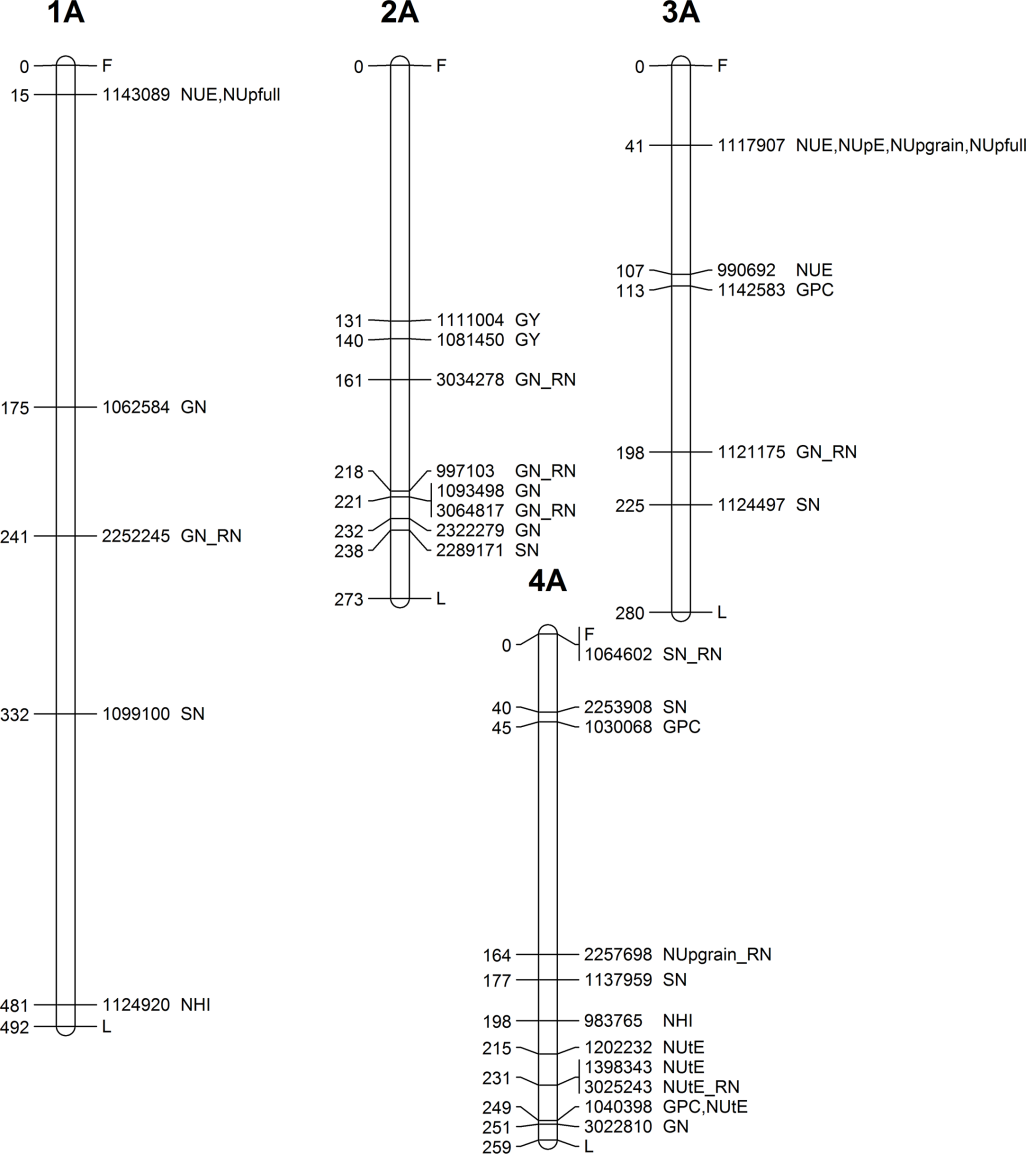
Genetic map showing the chromosomal locations of MTAs on chromosomes 1A, 2A, 3A and 4A. Map distances (cM) are presented on the left side, while the corresponding marker ID and the type of trait are listed on the right side of the chromosome.

**Fig 4:**
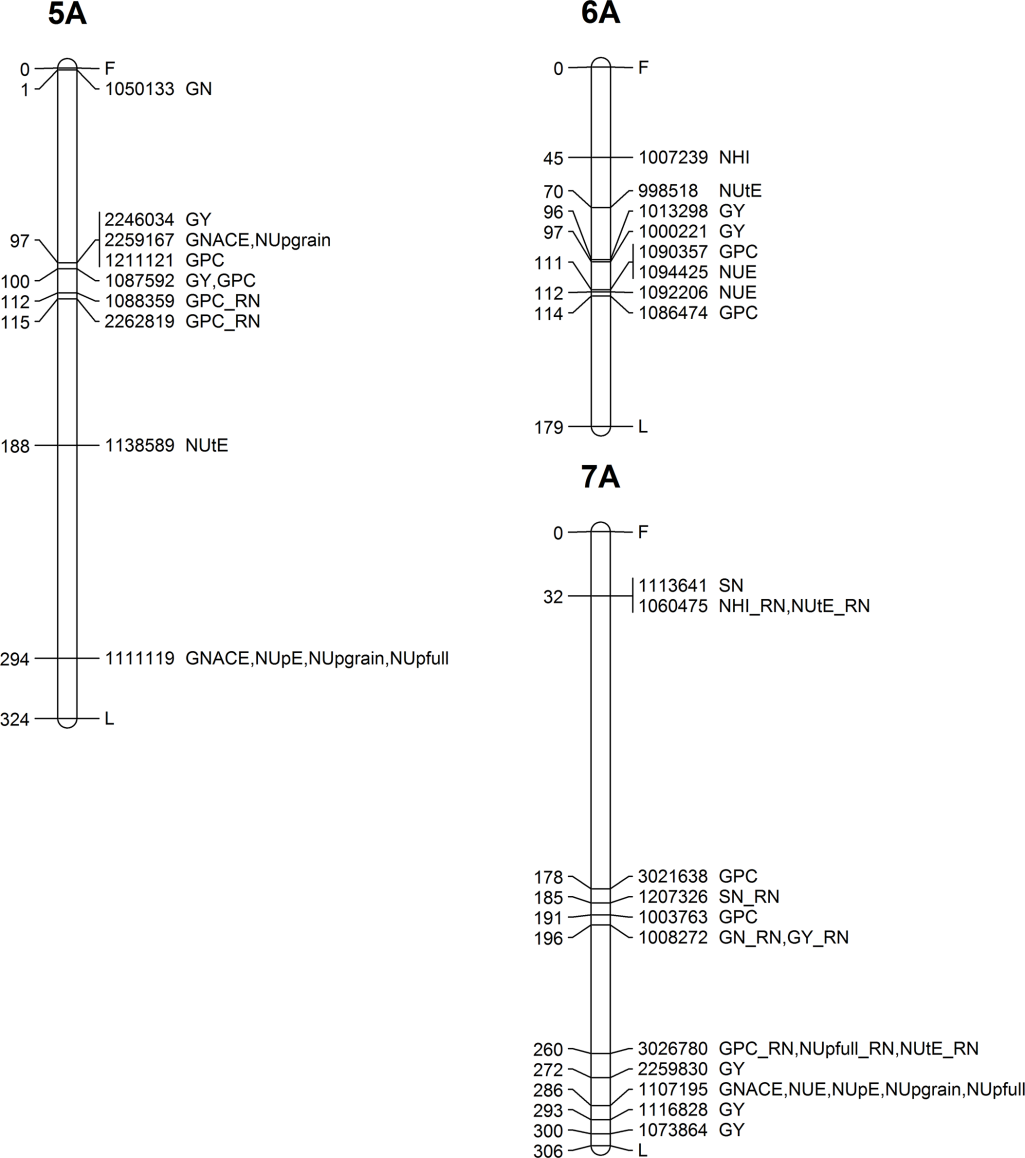
Genetic map showing the chromosomal locations of MTAs on chromosomes 5A, 6A and 7A. Map distances (cM) are presented on the left side, while the corresponding marker ID and the type of trait are listed on the right side of the chromosome.

**Fig 5:**
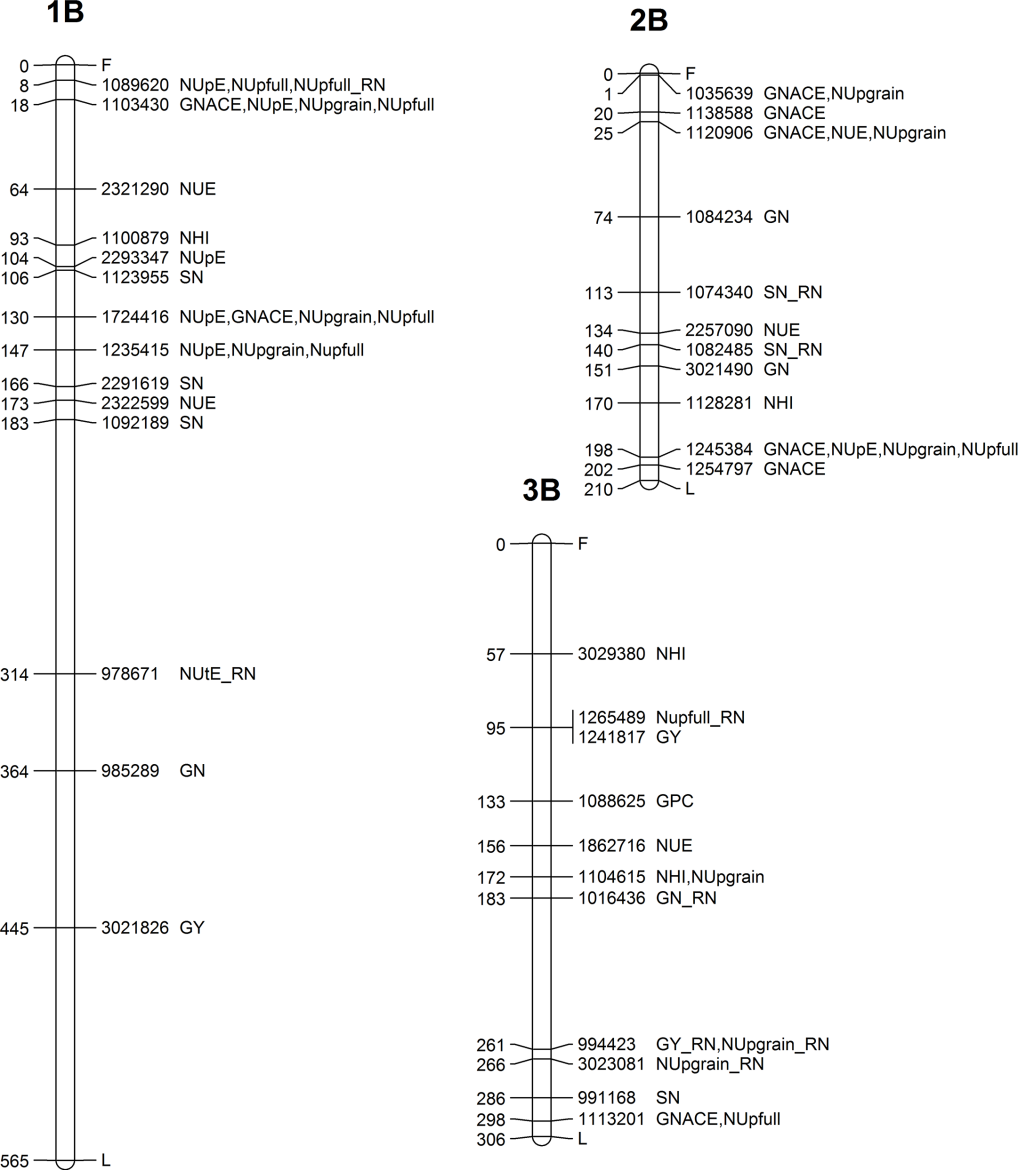
Genetic map showing the chromosomal locations of MTAs on chromosomes 1B, 2B and 3B. Map distances (cM) are presented on the left side, while the corresponding marker ID and the type of trait are listed on the right side of the chromosome.

**Fig 6:**
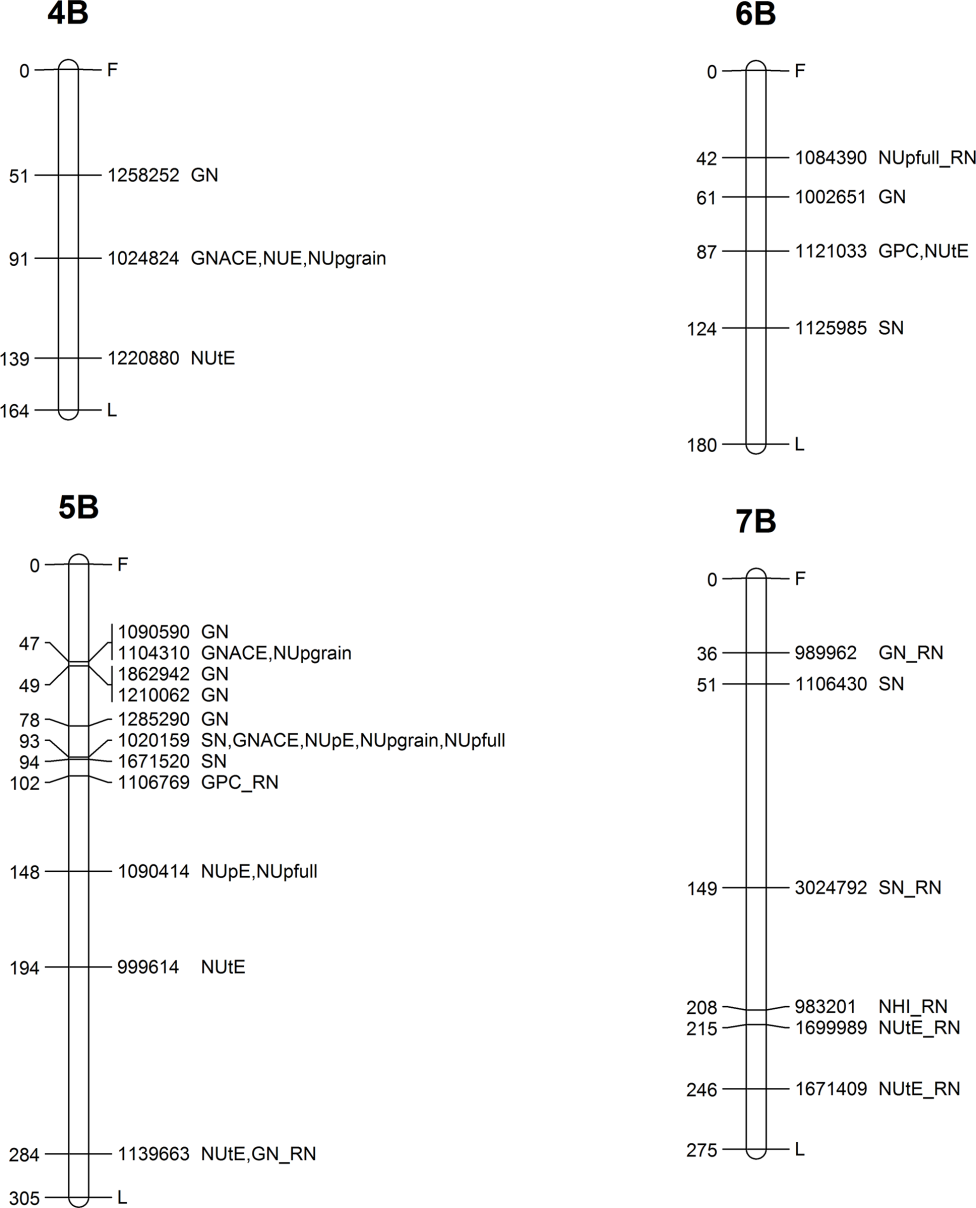
Genetic map showing the chromosomal locations of MTAs on chromosomes 4B, 5B, 6B and 7B. Map distances (cM) are presented on the left side, while the corresponding marker ID and the type of trait are listed on the right side of the chromosome.

**Fig 7:**
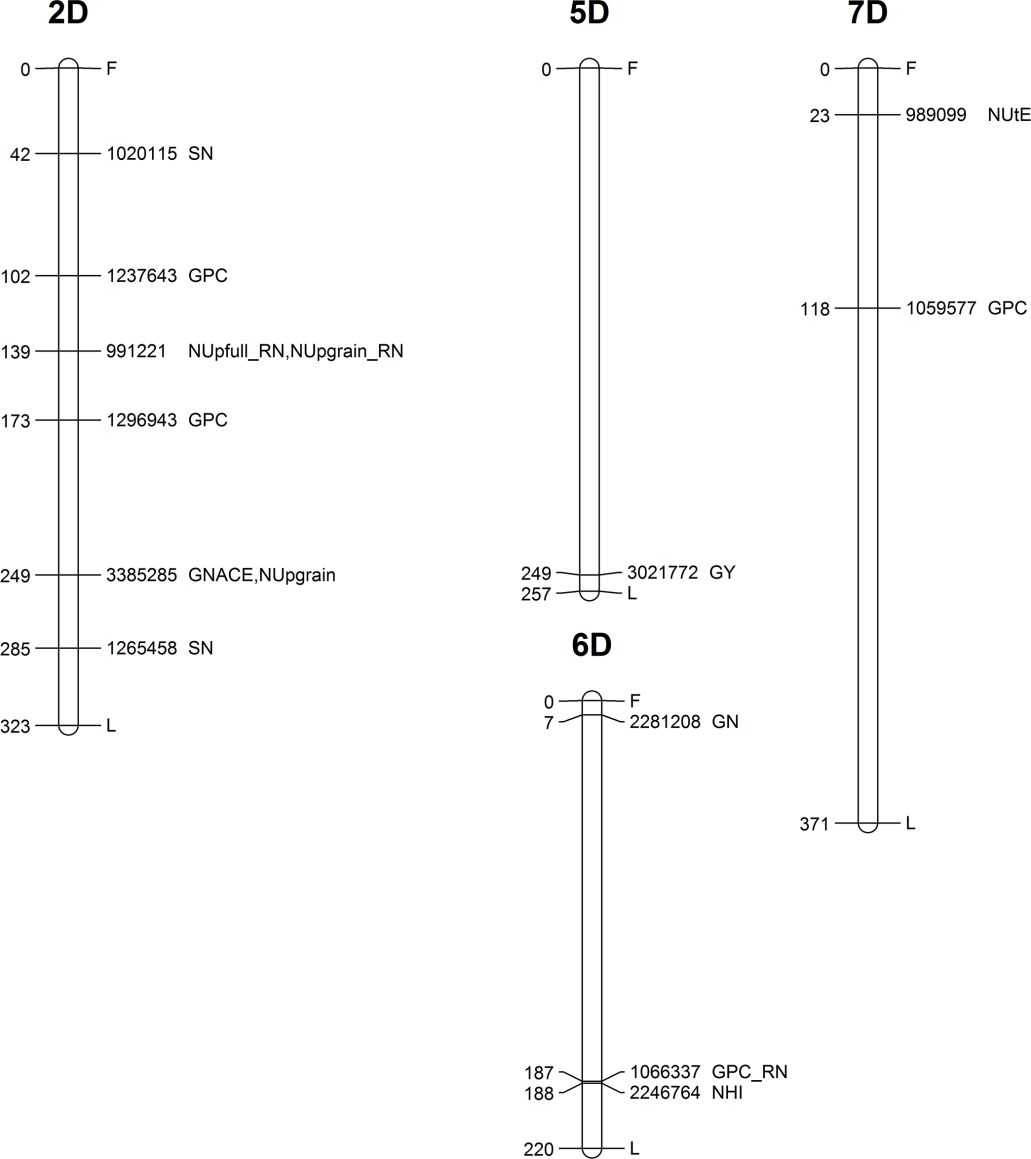
Genetic map showing the chromosomal locations of MTAs on chromosomes 2D, 5D, 6D and 7D. Map distances (cM) are presented on the left side, while the corresponding marker ID and the type of trait are listed on the right side of the chromosome.

Genome-wide association analysis was performed for 11 investigated traits (GY, NUE, NUpE, NUtE, NUp_full_, NUp_grain_, GPC, NHI, GNACE, GN, SN) in all environments (three cropping seasons, two N fertilisation levels) and for the response to N fertilisation for 8 selected traits in order to identify genomic regions involved in the response to N fertilisation. Altogether, 183 MTAs were identified for 130 DArTseq® markers. Most chromosomes were involved in determining at least one trait except chromosome 1D and 3D. All the details of the significant MTAs defined above are given in Table 3, while their chromosomal location is presented in Figs 3–7. SN was involved in the highest number of MTAs (17 MTAs) followed by GPC trait (16 MTAs), while for GY_RN and NHI_RN only 2 MTAs were identified. Altogether, the B genome offered the highest number of MTAs (93) followed by the A (75), while on the D genome only 15 MTAs were identified. Considering the homeologous groups, group 2, 5 and 1 contained the highest number of MTAs (34, 33 and 31), while lower number of MTAs were found on groups 3 and 7 (22 and 27). Chromosome group 4 and 6 had the lowest number of significant MTAs (18) among all traits.

**Table 3:** List of all significant MTAs identified under untreated (N0) or N-treated (N120) circumstances (A) and MTAs identified as N-response in selected traits (B).

**A**							
**Trait**	**Year**	**Chr**	**Position (cM)**	**Marker name**	**-log10 (p-value)**	**Effect**	**N input level**
GY (kg ha^-1^)	2014	1B	445	3021826	4.66	551.39	0
	2014	1B	445	3021826	2.96	347.67	120
	2015	2A	131	1111004	3.28	-767.75	120
	2015	2A	140	1081450	3.40	-152.90	120
	2014	3B	95	1241817	3.13	559.89	0
	2013	5A	97	2246034	3.08	673.98	120
	2013	5A	100	1087592	2.71	-255.72	120
	2013	5D	249	3021772	3.21	-549.87	120
	2014	6A	96	1013298	2.62	274.96	0
	2014	6A	97	1000221	2.76	580.40	120
	2014	7A	272	2259830	2.88	37.97	120
	2014	7A	293	1116828	2.86	-263.19	0
	2014	7A	300	1073864	3.48	182.54	120
Spike number per m	2015	1A	332	1099100	3.09	-14.85	0
	2015	1B	106	1123955	2.88	-12.59	120
	2013	1B	166	2291619	3.07	10.36	0
	2014	1B	183	1092189	3.63	13.32	0
	2014	2A	238	2289171	3.64	11.08	120
	2014	2D	42	1020115	2.80	-8.17	0
	2015	2D	285	1265458	2.88	1.88	120
	2013	3A	225	1124497	3.31	-10.14	120
	2015	3B	286	991168	2.92	-11.71	0
	2015	4A	40	2253908	3.24	-19.70	120
	2013	4A	177	1137959	2.91	-9.27	120
	2015	5B	93	1020159	3.39	13.22	0
	2015	5B	94	1671520	3.03	-11.69	120
	2015	6B	124	1125985	3.11	-13.99	0
	2015	6B	124	1125985	3.31	-15.69	120
	2014	7A	32	1113641	2.66	-9.20	0
	2014	7B	51	1106430	2.82	-9.51	120
Grain number per spike	2014	1A	175	1062584	2.62	5.35	0
	2014	1B	364	985289	2.73	3.71	120
	2015	2A	221	1093498	2.68	3.21	120
	2014	2A	232	2322279	3.03	-3.95	0
	2013	2B	74	1084234	3.03	-3.73	120
	2014	2B	151	3021490	2.83	4.29	120
	2013	4A	251	3022810	3.29	5.00	120
	2014	4B	51	1258252	3.72	5.42	120
	2013	5A	1	1050133	3.05	3.53	120
	2015	5B	47	1090590	2.86	3.18	0
	2015	5B	49	1210062	3.02	3.33	0
	2015	5B	49	1862942	3.53	3.68	0
	2013	5B	78	1285290	4.11	-4.80	120
	2013	6B	61	1002651	2.72	4.26	120
	2014	6D	7	2281208	2.76	-3.97	0
GNACE	2014	1B	18	1103430	2.96	-0.05	0
	2014	1B	130	1724416	3.12	-0.06	0
	2013	2B	1	1035639	3.47	-0.34	0
	2013	2B	20	1138588	3.05	0.31	0
	2015	2B	25	1120906	3.23	-0.13	120
	2013	2B	198	1245384	2.78	-0.13	120
	2014	2B	202	1254797	3.01	0.03	120
	2015	2D	249	3385285	3.04	-0.32	0
	2014	3B	298	1113201	2.74	-0.05	0
	2014	4B	91	1024824	2.70	-0.03	120
	2014	5A	97	2259167	2.65	0.05	0
	2014	5A	294	1111119	3.58	-0.05	0
	2014	5B	47	1104310	2.63	0.03	120
	2015	5B	93	1020159	2.64	0.19	0
	2015	7A	286	1107195	3.17	-0.14	120
GPC (w/w %)	2015	2D	102	1237643	3.14	-1.04	120
	2013	2D	173	1296943	2.74	1.23	120
	2014	3A	113	1142583	3.65	-1.42	0
	2013	3B	133	1088625	2.70	1.35	120
	2015	4A	45	1030068	3.13	0.90	120
	2014	4A	249	1040398	2.81	1.55	0
	2013	4D	90	1111560	2.83	-1.14	120
	2015	5A	97	1211121	3.28	0.84	0
	2015	5A	100	1087592	3.49	1.02	0
	2015	6A	111	1090357	2.75	-0.79	0
	2015	6A	114	1086474	2.65	-0.67	0
	2014	6B	87	1121033	3.45	-1.29	120
	2015	6B	87	1121033	2.76	-0.88	120
	2015	7A	178	3021638	3.02	0.72	120
	2015	7A	191	1003763	2.70	-0.90	0
	2015	7D	118	1059577	3.02	0.88	120
NHI	2015	1A	481	1124920	2.93	0.05	0
	2014	1B	93	1100879	3.25	0.08	0
	2015	2B	170	1128281	2.90	0.04	120
	2014	3B	57	3029380	2.73	0.08	0
	2014	3B	172	1104615	3.62	0.09	0
	2015	4A	198	983765	3.06	0.04	0
	2015	6A	45	1007239	3.10	0.04	0
	2014	6D	188	2246764	2.85	0.06	0
NUE	2013	1A	15	1143089	3.85	-7.96	120
	2014	1B	64	2321290	3.22	2.03	0
	2014	1B	173	2322599	2.68	-1.68	120
	2015	2B	25	1120906	3.35	-5.72	120
	2014	2B	134	2257090	3.47	2.13	0
	2014	2B	134	2257090	2.75	1.56	120
	2014	3A	41	1117907	2.98	2.02	0
	2014	3A	107	990692	2.67	1.84	0
	2013	3B	156	1862716	2.86	-15.52	0
	2014	4B	91	1024824	2.91	-1.72	120
	2015	6A	111	1094425	3.59	13.24	0
	2015	6A	112	1092206	3.53	10.82	0
	2015	7A	286	1107195	2.91	-5.62	120
NUpE	2014	1B	8	1089620	3.44	-0.07	0
	2014	1B	18	1103430	3.39	-0.07	0
	2014	1B	104	2293347	3.02	-0.06	0
	2014	1B	130	1724416	3.41	-0.08	0
	2014	1B	147	1235415	3.38	-0.08	0
	2013	2B	198	1245384	2.92	-0.16	120
	2014	3A	41	1117907	3.20	0.05	0
	2014	5A	294	1111119	3.31	-0.06	0
	2015	5B	93	1020159	3.06	0.30	0
	2014	5B	148	1090414	2.71	-0.05	120
	2015	7A	286	1107195	3.15	-0.16	120
NUtE	2014	4A	215	1202232	2.77	-8.41	0
	2014	4A	231	1398343	3.48	-12.84	0
	2014	4A	249	1040398	3.04	-5.80	120
	2014	4B	139	1220880	3.04	-10.12	0
	2014	5A	188	1138589	3.44	-5.44	120
	2014	5B	194	999614	3.10	5.88	120
	2014	6B	87	1121033	3.02	5.60	120
	2014	7D	23	989099	2.80	8.73	0
	2015	5B	284	1139663	2.64	2.56	120
	2015	6A	70	998518	3.00	2.92	0
NUp_grain_	2013	2B	1	1035639	3.06	-6.71	0
	2013	2B	198	1245384	2.78	-17.86	120
	2014	1B	18	1103430	3.03	-24.32	0
	2014	1B	130	1724416	3.16	-30.22	0
	2014	1B	147	1235415	3.06	-28.54	0
	2014	3A	41	1117907	3.19	21.83	0
	2014	3B	172	1104615	2.69	25.73	120
	2014	4B	91	1024824	2.69	-19.49	120
	2014	5A	97	2259167	2.63	23.61	0
	2014	5A	294	1111119	3.65	-24.48	0
	2014	5B	47	1104310	2.66	18.24	120
	2015	2B	25	1120906	3.23	-25.40	120
	2015	2D	249	3385285	3.04	-24.92	0
	2015	5B	93	1020159	2.64	14.77	0
	2015	7A	286	1107195	3.17	-26.76	120
NUp_full_	2013	1A	15	1143089	2.66	-21.38	120
	2013	2B	198	1245384	2.80	-21.96	120
	2014	1B	8	1089620	3.28	-30.25	0
	2014	1B	18	1103430	3.28	-30.00	0
	2014	1B	130	1724416	3.50	-37.62	0
	2014	1B	147	1235415	3.43	-35.78	0
	2014	3A	41	1117907	3.09	24.94	0
	2014	3B	298	1113201	3.04	-29.60	0
	2014	5A	294	1111119	3.33	-26.64	0
	2014	5B	148	1090414	2.69	-29.79	120
	2015	5B	93	1020159	3.06	23.01	0
	2015	7A	286	1107195	3.14	-32.31	120
**B**							
GN_RN	2013	2A	221	3064817	3.13	0.375235	nr
	2014	2A	161	3034278	3.02	0.150963	
	2014	2A	218	997103	4.39	-0.21002	
	2014	3B	183	1016436	2.97	-0.21002	
	2014	5B	284	1139663	4.21	-0.17151	
	2015	1A	241	2252245	2.69	-0.1474	
	2015	3A	198	1121175	3.32	-0.22085	
	2015	7A	196	1008272	2.82	-0.15467	
	2015	7B	36	989962	3.26	-0.15765	
GPC_RN	2013	7A	260	3026780	2.77	0.178959	nr
	2014	5A	112	1088359	3.79	0.092405	
	2014	5A	115	2262819	3.12	0.081973	
	2014	6D	187	1066337	2.96	-0.10421	
	2015	5B	102	1106769	2.89	0.070948	
GY_RN	2014	3B	261	994423	2.98	-0.35425	nr
	2015	7A	196	1008272	3.04	-0.28011	
NHI_RN	2014	7B	208	983201	3.02	-0.08743	nr
	2015	7A	32	1060475	3.05	-0.11334	
NUp_full__RN	2013	3B	95	1265489	2.67	-0.70628	nr
	2013	7A	260	3026780	2.92	0.538769	
	2014	1B	8	1089620	2.85	0.333618	
	2014	6B	42	1084390	2.74	-0.31897	
	2015	2D	139	991221	3.02	0.3129	
NUp_grain__RN	2013	4A	164	2257698	2.81	-0.82981	nr
	2014	3B	261	994423	3.03	-0.38351	
	2014	3B	266	3023081	3.27	-0.34859	
	2015	2D	139	991221	2.87	0.324298	
NUtE_RN	2013	7A	260	3026780	4.58	-0.18675	nr
	2014	1B	314	978671	2.94	0.146016	
	2014	7B	215	1699989	3.04	0.062906	
	2015	4A	231	3025243	3.05	0.010906	
	2015	7A	32	1060475	3.68	-0.1294	
	2015	7B	246	1671409	2.70	-0.10625	
SN_RN	2013	2B	113	1074340	2.76	0.164955	nr
	2013	2B	140	1082485	3.08	0.169218	
	2015	4A	0	1064602	2.88	0.153547	
	2015	7A	185	1207326	3.64	-0.26287	
	2015	7B	149	3024792	3.15	0.201957	

Abbreviations: grain yield (GY), spike number (SN), grain number (GN), grain nitrogen accumulation efficiency (GNACE), grain protein content (GPC), harvest index (HI), nitrogen harvest index (NHI), nitrogen use efficiency (NUE), nitrogen uptake efficiency (NUpE), nitrogen utilization efficiency (NUtE), nitrogen taken up by yield (NUp_grain_), N taken up by the whole above ground plant (NUp_full_). Response to N level assessed for GY (GY_NR), response to N level assessed for GN (GN_NR), response to N level assessed for GPC (GPC_NR), response to N level assessed for NHI (NHI_NR), response to N level assessed for SN (SN_NR), response to N level assessed for NUtE (NUtE_NR), response to N level assessed for NUp_full_ (NUp_full_ _NR), response to N level assessed for NUp_grain_ (NUp_grain_ _NR), not relevant (nr). The estimated allele effect (Effect) is the corresponding metric for each trait.

## Discussion

### Characterisation of the effects of different environments, fertilisation and genotypes

The analysis of variance revealed that the environmental effect was the main factor controlling most of the studied traits (S4 Table). Strong interaction with the environment poses major challenge to developing N-related traits [43]. “Environmental effect” in the actual experiment indicates not just the considerable effect of the weather conditions but it also interprets the differences in the soil N supply. Although, the soil type at each location was chernozem, the average N_mineral_ content significantly differed between years: it was 21, 494 and 78 kgha^-1^ in 2013, 2014 and 2015, respectively. Not surprisingly, the environmental effect (including the effect of different N_mineral_) had greater influence and caused larger differences for most of the traits than the input level itself (120 kgha^-1^ N fertiliser) (S4 Table; S2 Table).

We found that the input level was not significant for HD; moreover, in the case of HI and TGW, the “genotype effect” explained the highest proportion of the phenotypic variance (S4 Table). These traits are known to be highly influenced by genetic factors: the highly heritable traits are mainly controlled by the genotype, and the high heritability of these traits is well documented [44–45]. Genotype explained the second highest proportion of the phenotypic variance for most of the traits (GY, NUtE, NHI, HD, PH, SN, GN and GPC) which indicates existing genetic variability in the population to improve these traits.

The effect of the input level was significant for most of the traits except TGW, HD (S4 Table), and it explained the second highest proportion of the phenotypic variance for NUE, GNACE, NUpE, NUp_full_ and NUp_grain_. NUE; GNACE and NUpE values were influenced by the amount of N available for plant. Unfortunately, the accurate determination of the amount of N available for plant is very difficult because it is very expensive, time-consuming and labour-intensive to accurately determine N losses and immobilisation through the whole season. Moreover, it has been proved that all the applied N is never available for the plant [10]. Therefore, while interpreting these traits, we have to account for N losses; otherwise the result can be misleading. For example, the decreasing efficiency at the higher N supply was not necessarily due to decreased plant efficiency but it was mostly because of the greater losses from the system. N fertilisation significantly increased GY and its components (GN and SN) in all seasons (S2 Table). HI was significantly increased by the N treatments in 2012–2013 and 2014–2015. Wheat crops generally respond to the increased N rates with higher HI until an optimum N supply is reached. Then the excessive N application leads to a decline in HI [46]. Since we did not find such decline in population level, it can be presumed that the N fertilisation was not excessive for most of the cultivars.

As an overall consideration, it can be concluded that the “genotype × environment” interaction caused important contribution to variation for most of the traits in our study, indicating that genotypes reacted differently to varying environmental conditions.

### Relationship between the parameters

Correlation coefficients between NUE and its components revealed that NUpE explained much more of the variation than NUtE in all seasons (Table 2). Our results are consistent with previous studies which declared that contributions of NUpE and NUtE to the overall NUE depends mostly on the level of N supply. When the available N is rare, the ability to absorb N is more important, which is mostly related to root characteristics. On the contrary, at high N levels, variation in NUE is mainly due to differences in NUtE. Accordingly, when N uptake is not limited by N_soil_ or climatic conditions, NUtE and HI become more important to produce higher yield.

In our current study, GPC showed negative correlation with NUE, GY and NUtE, while GPC showed mostly no significant correlation with NHI (Table 2). The inverse relationship between GPC and NUtE in winter wheat is well known. If GY is increased at fixed NUpE, it can cause a decline of GPC, unless NHI is increased [15]. In practice, unfortunately, the simultaneous increase of NHI and GY is very difficult. It was highlighted by [15] that a better post-anthesis-N-remobilization is important to increase NHI, nevertheless a high GY is remarkably depending on canopy photosynthesis, which requires a maintained minimal leaf N concentration. The early transfer of photosynthetic N from leaves and stem would lead to a reduced photosynthesis and, as a consequence, to a reduced GY. Therefore, optimisation of timing and spatial patterns of senescence and N remobilisation is desired to increase yield while maintaining GPC.

The analysis of variance revealed that in the years 2012-2013 and 2014-2015 the variation in GN explained most of the variations in GY followed by SN, while in 2013-2014 an opposite tendency was observed. Correlations of NUE (GY) with GN and SN over cropping seasons confirmed the previously mentioned results (Table 2). It was described, that SN is determined earlier in the growing season than GN and TGW [47]. This period precedes the double ridge stage and it ends at booting stage. In the year 2013-2014 the high N_soil_ and the good soil moisture content increased the spike numbers by a greater production of tillers initially and/or by a higher percentage of tillers that survived to form spikes at maturity. Since we have not monitored tillering, it is not possible to establish which one affected the spike number more effectively. The year 2012-2013 was characterised by low N_mineral_ with severe drought after tillering and probably this complex effect led to the decreased spike number. In the last cropping season (2014-2015) the rainy winter and the mild spring stimulated the vegetative development and the forthcoming spike formation.

GN is determined firstly by the number of spikelets and then by the number of florets that produce grain. Therefore, N deficiency can affect GN during a long period lasting from the double ridge stage to the anthesis. In the year 2013-2014, not just the SN but the GN was also high, indicating a high N_mineral_ and an ideal soil moisture condition. In the 2014-2015 growing season the SN was high, however, low TGW and GN were measured since a drought period began in the spring, which greatly reduced the number of grains per spike.

TGW explained only a minor variation in GY. It showed a slightly negative correlation with SN and GN in most of the seasons as reported by other authors too [46]. It is not surprising, since TGW is mainly dependent on carbohydrate availability, rather than N assimilate supply [16]. SN and GN are determined earlier than TGW. If no sufficient assimilation occurs in the late growing season, the high total grain number (determined earlier in the season) may lead to assimilate partitioning, thus the TGW may decrease.

### Distribution of the markers among genomes, population structure and linkage disequilibrium

Among the polymorphic markers, 37% were located on the A, 50% on the B and only 13% on the D genome. A very similar distribution of the polymorphic DArT and DArTseq® markers were observed in bread wheat collections [38,44,48]. Previous studies reached the conclusion, that larger proportion of genetic diversity is originated from the tetraploid ancestor of hexaploid wheat, much more than from *Aegilops tauschii*, the source of the D genome [49]. Other studies concluded that high marker density is needed to capture the existing variation for gene localisation on the D genome [17]. In our current work the 3,290 polymorphic mapped markers were sufficient to explore the whole genome with the exception of some chromosomes on the D genome.

Two subpopulations (Sp1 and Sp2) were identified with the Bayesian approach implemented in Structure [32] and confirmed by PCoA. The majority of the 79 cultivar belonged to Sp1, while Sp2 incorporated the remaining 14 cultivars. This structure is consistent with the breeding history of the cultivars. Cultivars in Sp1 mainly originated from Central-Europe and Hungary, while the cultivars in Sp2 descended from Mironovskaya and/or Mv Magdaléna varieties, these cultivars in Sp2 were marketed between 1996 and 2009 by Elitmag Ltd. Martonvásár, Hungary. Separate analysis of chromosomes suggested that different selective pressures may act on chromosomes 1B and 2D between the two subpopulation (S3 Table), which may be related to the presence loci related with important agronomic traits for wheat breeding [50]. High number of pairs with complete LD and high number of linked pairs on chromosome 1B may be related with the 1BL.1RS wheat-rye translocation, which has been introduced in the areas of the former Soviet Union as a source of resistance against foliar fungal diseases through the widely used Russian varieties Bezostaya-1, Aurora and Kavkaz [51–53]. Large number of wheat varieties has been developed from crosses involving these varieties. Moreover, these high-yielding, early-maturing, semi-dwarf wheat varieties also carry the *Reduced height 8* (*Rht8)* dwarfing alleles on chromosome 2D [54]. In Hungary the two main breeding programs introduced different sources of resistance against foliar fungal diseases. In Martonvásár (Elitmag Ltd.,) the above mentioned Russian cultivars were frequently used; therefore, the 1BL.1RS wheat-rye translocation played a dominant role. Their progeny cultivar Mv Magdaléna was used for a long period to integrate the 1BL.1RS translocation into the novel varieties [52–53]. On the other hand, in the second main Hungarian breeding company (Cereal Research Nonprofit Ltd., Szeged, Hungary) the *Sr36/Pm6* gene cluster was applied at high frequency. The high mean r^2^ and high number of linked marker pairs we observed on chromosome 2D might be the result of *Rht8* locus alleles. *Rht8* dwarfing alleles on chromosome 2D is widely used in Central-Europe to reduce final plant height [38].

Moreover, under Hungarian environmental conditions (a temperate continental environment), the *PPD-D1* gene, located on chromosome 2D, has the largest genotypic effect on heading date [55]. Timing of flowering is an important component of adaptation in continental climate to improve grain filling before the onset of desiccating summer conditions. In a world-wide wheat collection of 683 genotypes [55] investigated varieties from entire Europe, including genotypes from our collection. In this study allele frequencies for vernalisation response (*VRN-A1*, *VRN-B1*, *VRN-D1*) and for photoperiod sensitivity (*PPD-B1*, *PPD-D1*) were investigated. It was determined that the two most frequent allele combinations among the Central European genotypes carry the recessive (winter) allele for all three *VRN1* genes with the sensitive allele type of the *PPD-B1* gene, while they differed in *PPD-D1*. Therefore, the photoperiod-sensitive allele, *PPD-D1* must also contribute the observed difference in LD on chromosome 2D between two subpopulations in our experiment.

We studied different aspects of LD in our panel. An average LD decay distance for our total population was found to be 9 cM. Numerous studies in wheat have reported LD decay distances; although, those reported LD decay distances vary greatly between studies. A wheat collection from Northern Europe was characterised by [44] on wheat DArTseq® platform and the average intra-chromosomal LD decay was calculated separately for SNP markers and silicoDArT markers. The average LD decay of the whole genome was observed to be at 20.59 and 9.51 cM for silicoDArTs and SNPs, respectively. Recently, an LD decay distance of 23 cM was observed for 94 European bread wheat genotypes using 1,849 genome-wide distributed DArT markers [38]. In another DArT marker-based study, 251 winter wheat lines displayed a narrower, 9.9 cM LD decay for the whole genome [56]. These differences may be caused by different genetic variability and dissimilar population size between the populations. In line with previous results [38] we found the highest LD on the D genome.

### Marker trait associations

Here we describe the genetic characterisation of NUE and several agronomically relevant traits. Moreover we also report the identified genetic loci involved in the response to input level under different N fertiliser regimes. In general, B genome showed the highest number of MTAs (93) and it was followed by the A genome (75), while only 15 MTAs were found on the D genome. Among the homologous chromosomes, group 2, 5 and 7 contained the most significant associations, especially on 1B and 7A chromosomes (25 and 19 MTAs, respectively). Particularly, chromosome 1B proved to be highly rich in MTAs, since this chromosome contains the 1RS.1BL translocation, which transfers a number of genes of agronomic importance. The positive and negative impact of the 1RS.1BL translocation on agronomically important traits is well studied [25,57].

Different numbers of MTAs were detected between cropping seasons in our experiment. Considering, that the soil N content and the weather parameters (amount and timing of precipitation, timing and duration of drought, hot periods) were quite different in every year, the three experimental seasons can be considered as three different environments. We found that most of the MTAs were environmental-dependent and also that most of the MTAs were detected in one environment only. These environment-specific MTAs reflect regions associated with adaptation to specific environmental conditions. Twenty-eight markers were associated with multiple traits (S5 Table). This situation occurs when high LD exists between loci for different traits or when the pleiotropy is the main cause of the genetic correlation between traits. Identifying multi-trait MTA can help to understand the physiological control of multiple traits. Despite this environmental variability, 4 DArTseq® markers were significant for the same trait in two different environments (detailed in Table 3). Three marker (clone ID 3021826, 2257090 and 1125985) were associated in both treatments in the same year for the same trait (GY, NUE and SN) indicating that they were not (or just partially) influenced by N fertilisation, while only one marker (clone ID 1121033) was significant for the same trait (GPC) in two different years. These genomic regions are the best candidates for more extensive studies because they may be involved in constitutive processes for agronomically important traits.

However in the previous works, the genes encoding the key enzymes of N metabolism and the QTLs determining their activities had been investigated mostly with SSR, SNP markers, hence it was not possible to co-localise and compare our results by these studies. Therefore, it would be useful integration of DArTseq® markers with other marker systems to identify next-generation sequencing markers, which are physically linked to the known major genes responsible for adaptation to environmental conditions and also those genes which are involved in the carbon and N metabolism. This would facilitate the screening of allelic variation in the breeding populations and help investigate the influence of the above mentioned major genes on agronomically important traits.

### MTAs for grain yield and its components

Complex traits like GY are regulated by a number of metabolic networks and many traits have a downstream effect on crop yield. Nine chromosomes were involved in the control GY and its most important components (GN, SN). Only few studies used GBS or DArT markers to investigate yield components, therefore it is difficult to align and compare our results by the earlier studies using different marker systems. Identified chromosomal regions affecting grain yield and its components are good candidates to be used in breeding programs to improve the performance of wheat varieties. 12 loci were identified that are involved in the variation of GY, and several of these MTAs correspond to QTLs already reported in other studies, using different wheat mapping populations. A QTL (*QYld-1B*) for GY was identified by [58] under drought conditions on chromosome 1B. In the same region an MTA (clone ID 3021826) was observed in our study. In a study by [59] two QTLs (*QGY*.*ndsu*.*2A*.*1* and *QGY*.*ndsu*.*2A*.*2*) were identified for GY which correspond to two MTAs (clone ID 1081450 and 1111004) we located on the same position on chromosome 2A. Two QTLs (*wPt-7901* and *wPt-6687*) were observed by [25] for GY on chromosome 2A, where two MTAs (clone ID 2322279 and 2289171) were identified for main yield components (GN and SN) in the present study. Another MTA associated with GY was identified in the present study on chromosome 3B, close to the significant *wPt-4209* marker for GY described by [25]. In the present study MTA for GY (clone ID 1073864) was observed on 7AL; in the same region a DArT marker (*wPt-6495*) was identified earlier by [25]. Our study also introduces loci affecting GY and its components that have never been discovered previously.

SN is the most easily identifiable yield component. On 11 chromosomes, 17 markers were significantly associated with SN. With a single exception on chromosome 5B, only trait-specific MTAs were observed. Some of these MTAs hit genomic regions that were previously reported to affect SN. A significant DArT marker (*wPt-9277*) for SN identified by [23] in close proximity to MTA (clone ID 2289171), observed in the present study on chromosome 2A. An MTA (clone ID 1113641) for SN is co-localised to the same region as a QTL (*QNS*.*ndsu*.*7A*) and the significant *wPt-5153* marker was observed on chromosome 7A by [23] and by [59]. Moreover, MTA associated with SN (clone ID 1137959) was identified in the present study on chromosome 4A, close to the significant *wPt-0817* marker [23].

Grain number per spike is an important yield component that has been studied extensively in the past. However, the identified QTLs were not consistent across treatments and cropping seasons, suggesting that the environment had considerable effect on the expression of the genes associated with GN. 15 markers were found to be significantly associated with GN and spread over 10 chromosomes. A QTL (on *wPt-9793* marker) for GN was identified [23] in close proximity to an MTA (clone ID 2322279) observed in the present study on chromosome 2A. A QTL (interval between *wPt-8604* and *wPt-5175*) for grains per square meter was identified on chromosome 5B by [60]. In our study MTAs affecting GN were mapped in this region. An another MTA (clone ID 1285290) associated with GN was identified in the present study on chromosome 5B, which coincide with the significant marker *wPt-6726* identified earlier by [23].

### MTAs for NUE and N-related traits

Each step contributing to NUE, i.e. the uptake, translocation, assimilation, and remobilization of N, is controlled by multiple interacting genetic and environmental factors. Most of the MTAs for NUE and its components were environment-specific in our study. We identified 12 significant DArTseq® markers for NUE on chromosomes 1A, 1B, 2B, 3A, 3B, 4B, 6A and 7A, across all treatments and cropping seasons. The identified MTAs will facilitate the exploration of the possible structural or regulatory genes controlling NUE under different, specific environmental conditions. Previous studies revealed the importance of NUpE in NUE improvement [11,13]. In our study efforts were made to investigate not just NUpE but the total amount of N accumulated in the above ground biomass (NUp_full_).

Genomic regions identified for NUp_full_ by [7], for N uptake by [18] and [19] and for NUpE and NUtE by [61] overlap with regions identified in our study, suggesting that some related regions may be significant. We found 11 markers significantly associated with NUpE and spread over 6 chromosomes (1B, 2B, 3A, 5A, 5B, 7A). Usually these markers were also significant for NUp_full_, NUp_grain_ and GNACE N-related traits. Likewise in previous studies, chromosome 1B was found to be rich in (5) MTAs for NUpE. In our study 12 NUp_full_ MTAs were presented on chromosomes 1A, 1B, 2B, 3A, 3B, 5A, 5B and 7A. Significant markers for NUp_full_ were related to more than one trait in most cases; besides NUp_full,_ they were also significant for NUpE and GNACE. 8 MTAs occurred in the extensive management system, while under intensive management only 4 MTAs were found.

N utilisation is the other important component of NUE, depending on both N assimilation and remobilisation [22]. In our study 10 MTAs were spread along on 7 chromosomes for NUtE. Most markers were trait-specific, while the remaining markers were significant with GPC. This might to be expected, since an increase of NUtE could reduce grain protein concentration. In our study 8 markers were identified for NHI as significant; altogether, 7 chromosomes were involved. Previous studies identified several QTLs for GPC in winter wheat [23, 47, 62–64]. In our study a total of 16 MTAs for GPC were detected across treatments and cropping seasons and the significant markers were spread along 10 chromosomes. Most of these trait-specific MTAs occurred in the vicinity of markers significant for NUtE or yield-related traits. Our findings may help to maintain or improve GPC under various N-conditions.

In our study differences in the ability to increase the harvested grain N content was also investigated (NUp_grain_). Moreover, the efficiency of the plant to take up, translocate and incorporate N into grain protein was also examined: GNACE measures the overall efficiency with which plants extract N from the soil and accumulate it in the grain [10]. 15 markers were found to be significantly associated with GNACE and NUp_grain_, spreading over 8 and 9 chromosomes. In most cases these two traits showed significant association on the same markers. Furthermore, usually the same markers were also significant for N uptake. The *wPt-1554* marker was in LD with *chloroplastic/plastidic glutamine synthetase* (*GS2*) gene on chromosome 2D [22], and in close proximity to this location several MTAs associated with GNACE and NUp_grain_ were identified in our study.

### MTAs for responsiveness to N fertilisation

Several significant QTLs have been detected in wheat under low N stress and normal N growth conditions which helped to partially understand the genetic control of NUE and related traits [7,20], although only few studies [18–19] characterised the responses of the agronomic traits to N fertilisation. A novel method, an association genetic study [22] used 3 computed parameters to characterize the response to N input level (for GY and grain number per m^2^): the difference and the ratio between performance at 2 N input levels and the slope of joint regression.

The present study provides an opportunity for the identification of MTAs induced by response to N fertilisation (RN). For such a characterisation, 8 traits (GY, SN, GN, GPC, NHI, NUp_full_, NUp_grain_ and NUtE) were expressed as the ratio between N120 and N0. The genomic regions involved in RN included 38 MTAs spread over 14 chromosomes (Table 3). Most of the identified RN MTAs do not overlap with regions identified for agronomically important traits in our study. Only 2 RN MTAs showed significant association with markers also linked to MTA for agronomic parameters identified in this study. Response to nitrogen level assessed for GN (GN_NR) showed significant association with 9 loci, for NUtE_NR with 6 loci; for GPC_NR, NUp_full__NR and SN_NR with 5 loci, for NUp_grain__NR with 4 loci and for GY_NR and NHI_NR with 2 loci. A large number of RN MTAs were detected on chromosome 7A, 7B and 3B. A DArT marker (*wPt-8330*) was found [22] to be involved in the response to N level on chromosome 2D where two MTAs (clone ID 991221) were identified for NUp_full__RN and NUp_grain__RN in the present study. In the same study two significant DArT markers (*wPt-1250* and *wPt-5153*) were identified for response to N level on chromosome 5B and 7A; in this position MTAs for GPC_RN and NHI_RN (clone ID 1106769 and 1060475) were found in our study.

## Conclusions

Here is presented a GWAS analysing NUE with its components and related agronomically relevant traits in a panel of commercially relevant Central European winter wheat cultivars genotyped on DArTseq® platform. In our current study the marker density was sufficient to identify MTAs almost genome, wide with the exception of some chromosomes of the D genome. So it was concluded that a higher polymorphic marker density is needed on the D genome to successfully reveal the genetic variation in all the homologous chromosomes. Since DArTseq® is a relatively new approach in cereal genetics, it was a challenge to compare our results with numerous earlier studies using different markers systems. Novel works, based on this marker system and the appearance of comparative maps will definitely facilitate such effort. In our study chromosomal regions involved in the regulation of N metabolism were localised on many chromosomes, many of them coinciding with major genes or QTLs detected in previous studies, indicating the precision of the association study. Furthermore, many DArTseq® markers were identified being significantly associated in regions where neither genes nor QTL had previously been reported for these traits.

The soil N content and the weather parameters were quite different in every year, therefore strong environmental effect and G × E interaction were observed for most of the examined traits. As a consequence most of the identified MTAs were environmental-dependent. These environment-specific MTAs may reflect regions associated with adaptation to specific environmental conditions. Since our cultivar collection is well-adapted to the Central European region. On the other hand, MTAs identified in the present study may be considered as first step for the understanding of the genetic basis of NUE and agronomically important traits. The determination of those genes by candidate gene identification efforts, which are anchored by markers identified by GWAS may help to explain such complex traits as nitrogen use efficiency at molecular level.

## Supporting information

S1 Table Wheat varieties investigated in the recent study. (DOCX)

S2 Table The average phenotypic values of the 15 traits measured in the whole panel and in the two subpopulations (Sp1 and Sp2), at two input levels (N0: 0 kg N hectare-1 and N120: 120 kg N hectare-1). Significant differences between the two treatments (A) and between the two subpopulations in the given traits (B) are indicated with asterisks.
(DOCX)

S3 Table Overview of intra-chromosomal LD on each chromosomes in the population. (DOCX)

S4 Table Results of analysis of variance for Genotype, Input level, Environment effect. (DOCX)

S5 Table Overview of markers associated with multiple traits. (DOCX)

S1 Fig Chromosome coverage of 3290 polymorphic and mapped DArTseq® markers. Map information was provided by Triticarte Pty. Ltd.
(TIF)

S2 Fig Phylogram of 93 winter wheat genotypes. (DOCX)

S3 Fig Q-Q plots of -log10 P-values for GWAS based on the MLM+K+Q model. (DOCX)

S4 Fig Graphs showing the phenotypic distribution of NUE in each year under extensive and intensive management. (DOCX)

## Abbreviations

AMOVA: analysis of molecular variance
GBS: genotyping by sequencing
G × E: genotype by environment interaction
GLM: general linear model
GN: grain number per spike
GNACE: grain N accumulation efficiency
GPC: grain protein content
GS: growth stage
GY: grain yield
GWAS: genome-wide association study
HD: heading date
HI: harvest index
LD: linkage disequilibrium
MLM: mixed linear model
MTA ATK: Centre for Agricultural Research, Hungarian Academy of Sciences
MTA: marker-trait association
N: nitrogen
N0: no nitrogen fertilisation, extensive management
N120: 120 kg per hectare nitrogen fertiliser, intensive management
NHI: N harvest index
N_mineral_: mineral N content
N_soil_: amount of N available in the soil and applied as fertiliser
NR: nitrogen response, trait_NR stands for the nitrogen response for the given trait
NUE: N use efficiency
NUpE: N uptake efficiency
NUp_full_: amount of N taken up by the whole aboveground plant
NUp_grain_: total N content harvested in the grain
NUtE: N utilisation efficiency
PCA: principal component analysis
PCoA: principal coordinate analysis
PH: plant height
QTL: Quantitative Trait Locus
RN: response to N fertilization
SN: spike number per meter
Sp: subpopulation
SY: straw yield
TGW: thousand grain weight
