# Supplementary material for: Genome-wide association study and genetic diversity analysis on nitrogen use efficiency in a Central European winter wheat (Triticum aestivum L.) collection
Source: PLoS One. 2017 Dec 28;12(12):e0189265. doi: 10.1371/journal.pone.0189265 (PMC5746223; doi:10.1371/journal.pone.0189265)
Supplement: S1 Table — (DOCX) [file pone.0189265.s001.docx]

**S1 Table. Wheat varieties with winter (W) or facultative (F) growth habits grown in Martonvásár, Hungary, during 2012‑2015.**

| **Cultivar** | **Country of origin** | **Growth habit** | **Year of release** | **Subpopulation** |
| --- | --- | --- | --- | --- |
| Josef | Austria | W | 1994 | 1 |
| Balaton | Austria | W | 2006 | 1 |
| Dunai | Austria | W | 2003 | 1 |
| Fulvio | Austria | W | 2009 | 1 |
| Yumai-34 | China | W | 1998 | 1 |
| Bakfis | Czech Republic | W | 2007 | 1 |
| Baletka | Czech Republic | W | 2007 | 1 |
| Bardotka | Czech Republic | W | 2004 | 1 |
| Kalahari | France | W | 2010 | 1 |
| Soissons | France | W | 1988 | 1 |
| Apache | France | W | 1998 | 1 |
| Euclide | France | W | 2007 | 1 |
| Mascot | France | W | 1977 | 1 |
| Betta | France | W | 1994 | 1 |
| Cordiale | Germany | W | 2005 | 1 |
| Florina | Germany | W | NA | 1 |
| GK Békés | Hungary, GKI^a^ | W | 2005 | 1 |
| GK Csillag | Hungary, GKI | W | 2005 | 1 |
| GK Fény | Hungary, GKI | W | 2005 | 1 |
| GK Futár | Hungary, GKI | W | 2011 | 1 |
| GK Garaboly | Hungary, GKI | W | 1998 | 1 |
| GK Göncöl | Hungary, GKI | W | 2009 | 1 |
| GK Hajnal | Hungary, GKI | W | 2010 | 1 |
| GK Hattyú | Hungary, GKI | W | 2002 | 1 |
| GK Kalász | Hungary, GKI | W | 1996 | 1 |
| GK Petur | Hungary, GKI | W | 1999 | 1 |
| GK Piacos | Hungary, GKI | W | 2003 | 1 |
| GK Rozi | Hungary, GKI | W | 2010 | 1 |
| GK Tisza | Hungary, GKI | W | 2003 | 1 |
| GK Verecke | Hungary, GKI | W | 1999 | 1 |
| GK Vitorlás | Hungary, GKI | W | 2010 | 1 |
| GK Berény | Hungary, GKI | W | 2010 | 1 |
| GK Kapos | Hungary, GKI | W | 2003 | 1 |
| GK Szala | Hungary, GKI | W | 2005 | 1 |
| GK Öthalom | Hungary, GKI | W | 1985 | 1 |
| GK Csongrád | Hungary, GKI | W | 2001 | 1 |
| GK Hunyad | Hungary, GKI | W | 2005 | 1 |
| GK Ati | Hungary, GKI | W | 2001 | 1 |
| Mv Apród | Hungary, MTA ATK^b^ | W | 2010 | 2 |
| Bánkúti 1201 | Hungary, MTA ATK | W | 1931 | 1 |
| Mv Béres | Hungary, MTA ATK | W | 2003 | 2 |
| Mv Bodri | Hungary, MTA ATK | W | 2008 | 1 |
| Mv Csárdás | Hungary, MTA ATK | W | 1999 | 2 |
| Mv Emese | Hungary, MTA ATK | W | 2000 | 1 |
| Mv Hombár | Hungary, MTA ATK | W | 2004 | 1 |
| Mv Karéj | Hungary, MTA ATK | W | 2011 | 1 |
| Mv Kolo | Hungary, MTA ATK | W | 2006 | 2 |
| Mv Kolompos | Hungary, MTA ATK | W | 2009 | 1 |
| Mv Ködmön | Hungary, MTA ATK | W | 2002 | 2 |
| Mv Lepény | Hungary, MTA ATK | W | 2011 | 1 |
| Mv Lucilla | Hungary, MTA ATK | W | 2007 | 1 |
| Mv Magdaléna | Hungary, MTA ATK | W | 1996 | 2 |
| Mv Magvas | Hungary, MTA ATK | W | 1998 | 1 |
| Mv Marsall | Hungary, MTA ATK | W | 2001 | 1 |
| Mv Mazurka | Hungary, MTA ATK | W | 2003 | 2 |
| Mv Menüett | Hungary, MTA ATK | W | 2009 | 2 |
| Mv Palotás | Hungary, MTA ATK | W | 2000 | 1 |
| Mv Pántlika | Hungary, MTA ATK | W | 2012 | 1 |
| Mv Pengő | Hungary, MTA ATK | W | 2012 | 1 |
| Mv Petrence | Hungary, MTA ATK | W | 2009 | 1 |
| Mv Regiment | Hungary, MTA ATK | W | 2004 | 1 |
| Mv Sobri | Hungary, MTA ATK | W | 2011 | 1 |
| Mv Suba | Hungary, MTA ATK | W | 2002 | 2 |
| Mv Süveges | Hungary, MTA ATK | W | 2002 | 2 |
| Mv Tallér | Hungary, MTA ATK | W | 2010 | 1 |
| Mv Toborzó | Hungary, MTA ATK | W | 2003 | 2 |
| Mv Toldi | Hungary, MTA ATK | W | 2008 | 2 |
| Mv Vekni | Hungary, MTA ATK | W | 2006 | 1 |
| Mv Nádor | Hungary, MTA ATK | W | 2012 | 1 |
| Mv Verbunkos | Hungary, MTA ATK | W | 2001 | 2 |
| Mv Zelma | Hungary, MTA ATK | W | 2008 | 1 |
| Mv Walzer | Hungary, MTA ATK | W | 2003 | 2 |
| Mv Karizma | Hungary, MTA ATK | F | 2009 | 1 |
| Mv Kikelet | Hungary, MTA ATK | F | 2010 | 1 |
| Mv Kokárda | Hungary, MTA ATK | W | 2012 | 1 |
| Karolinum | Netherlands | W | 2003 | 1 |
| Otilia | Romania | W | 2013 | 1 |
| Ostrov | Romania | W | NA | 1 |
| Pitar | Romania | W | NA | 1 |
| Jubilejnaja 50 | Russia | W | 1970 | 1 |
| Moskvich | Russia | W | 2006 | 1 |
| Starshina | Russia | W | 2003 | 1 |
| Krasnodarskaya-99 | Russia | W | 2003 | 1 |
| Bezostaja-1 | Russia | W | 1959 | 1 |
| IS Bonnet | Slovakia | W | 2008 | 1 |
| IS Karpatia | Slovakia | W | 2007 | 1 |
| IS Agape | Slovakia | W | 2009 | 1 |
| Kinaci-97 | Turkey | W | 1997 | 1 |
| Sultan95 | Turkey | W | 1995 | 1 |
| Konya | Turkey | W | 2002 | 1 |
| Slavna | Ukraine | W | 2010 | 1 |
| Nudakota | USA | W | 2006 | 1 |
| Hatcher | USA | W | 2004 | 1 |

Subpopulation numbers are defined by STRUCTURE analysis.

^a^ GKI: Cereal Research Nonprofit Ltd. (Szeged, Hungary)

^b^ MTA ATK: cultivar collection at MTA ATK (Martonvásár, Hungary)
