# Supplementary material for: Genome-wide association study and genetic diversity analysis on nitrogen use efficiency in a Central European winter wheat (Triticum aestivum L.) collection
Source: PLoS One. 2017 Dec 28;12(12):e0189265. doi: 10.1371/journal.pone.0189265 (PMC5746223; doi:10.1371/journal.pone.0189265)
Supplement: S2 Table — Significant differences between the two treatments (A) and between the two subpopulations in the given traits (B) are indicated with asterisks. (DOCX) [file pone.0189265.s002.docx]

**S2 Table. The average phenotypic values of the 15 traits measured in the whole panel and in the two subpopulations (Sp1 and Sp2), at two input levels (N0: 0 kg N hectare^-1^ and N120: 120 kg N hectare^-1^). Significant differences between the two treatments (A) and between the two subpopulations in the given traits (B) are indicated with asterisks.**

**A**

| **Trait** | **Group** | **2012-2013** | | **2013-2014** | | **2014-2015** | |
| --- | --- | --- | --- | --- | --- | --- | --- |
|  |  | **N0** | **N120** | **N0** | **N120** | **N0** | **N120** |
| Grain yield (kg hectare^-1^) | Population | 2338*** | 3951 | 4887*** | 6656 | 3802*** | 5139 |
|  | Sp1 | 2387*** | 4032 | 4842*** | 6597 | 3953*** | 5300 |
|  | Sp2 | 2012*** | 3420 | 5157*** | 7020 | 2882*** | 4157 |
| Straw yield (kg hectare^-1^) | Population | 3451*** | 4536 | 7380*** | 9013 | 6962*** | 8034 |
|  | Sp1 | 3491*** | 4582 | 7330*** | 8889 | 7083*** | 8087 |
|  | Sp2 | 3182*** | 4235 | 7681*** | 9764 | 6227** | 7708 |
| TGW (g) | Population | 38.1* | 39.8 | 44.0*** | 42.6 | 35.1 | 35.8 |
|  | Sp1 | 37.7** | 39.6 | 43.7*** | 42.4 | 34.9 | 35.7 |
|  | Sp2 | 41.4 | 41.2 | 45.5* | 43.5 | 36.3 | 36.6 |
| NUp_grain_ (kg N in GY) | Population | 35.6*** | 74.0 | 102.5*** | 145.7 | 83.0*** | 119.1 |
|  | Sp1 | 35.8*** | 75.3 | 100.2*** | 142.9 | 85.7*** | 122.1 |
|  | Sp2 | 34.6*** | 65.2 | 116.2*** | 162.7 | 66.0*** | 101.2 |
| NUp_full_ (kg N in biomass) | Population | 49.6*** | 91.6 | 129.8*** | 181.6 | 119.6*** | 164.5 |
|  | Sp1 | 50.0*** | 93.3 | 127.3*** | 178.3 | 123.1*** | 167.2 |
|  | Sp2 | 47.4*** | 80.5 | 144.4*** | 201.5 | 97.3*** | 147.6 |
| NUE | Population | 111.3*** | 28.0 | 9.9** | 10.8 | 48.7*** | 26.0 |
|  | Sp1 | 113.7*** | 28.6 | 9.8** | 10.7 | 50.7*** | 26.8 |
|  | Sp2 | 95.8*** | 24.3 | 10.4 | 11.4 | 36.9*** | 21.0 |
| NUpE | Population | 2.35*** | 0.64 | 0.26*** | 0.30 | 1.53*** | 0.83 |
|  | Sp1 | 2.36*** | 0.66 | 0.25*** | 0.29 | 1.57*** | 0.85 |
|  | Sp2 | 2.26*** | 0.56 | 0.29 | 0.33 | 1.25*** | 0.75 |
| NUtE | Population | 49.8*** | 45.5 | 39.9** | 37.0 | 31.7 | 31.1 |
|  | Sp1 | 50.7*** | 45.5 | 40.**5 | 37.3 | 32.2 | 31.7 |
|  | Sp2 | 44.5 | 45.4 | 36.3 | 35.4 | 28.9 | 27.9 |
| NHI | Population | 0.72*** | 0.81 | 0.80 | 0.80 | 0.70** | 0.72 |
|  | Sp1 | 0.72*** | 0.81 | 0.79 | 0.80 | 0.70*** | 0.72 |
|  | Sp2 | 0.72*** | 0.82 | 0.80 | 0.81 | 0.68 | 0.68 |
| Heading Date (day) | Population | 209.2 | 208.7 | 195.8 | 196.2 | 225.3 | 225.4 |
|  | Sp1 | 209.1 | 208.6 | 195.4 | 195.7 | 225.0 | 225.0 |
|  | Sp2 | 209.9 | 209.7 | 199.7 | 199.1 | 227.2 | 227.5 |
| Plant height (cm) | Population | 51.6* | 54.8 | 92.8*** | 95.7 | 74.9*** | 78.3 |
|  | Sp1 | 51.5* | 54.8 | 92.5** | 95.4 | 75.2** | 78.4 |
|  | Sp2 | 52.2 | 54.5 | 94.5** | 97.5 | 73.3** | 78.0 |
| Spike number per m | Population | 51.4** | 57.8 | 53.9*** | 66.8 | 71.0* | 82.9 |
|  | Sp1 | 53.4** | 58.7 | 54.5** | 67.2 | 72.4* | 83.6 |
|  | Sp2 | 44.8 | 51.5 | 50.3* | 63.5 | 61.8 | 78.7 |
| Grain number per spike | Population | 14.4*** | 21.0 | 25.1*** | 28.7^*^ | 18.6** | 21.0 |
|  | Sp1 | 14.6*** | 21.2 | 24.7*** | 28.3 | 19.0** | 21.6 |
|  | Sp2 | 13.0*** | 19.2 | 27.0* | 31.2 | 15.8 | 17.4 |
| Harvest Index | Population | 40.2*** | 46.0 | 39.5 | 42.4 | 35.1*** | 38.7 |
|  | Sp1 | 40.5*** | 46.4 | 39.4 | 42.5 | 35.7*** | 39.4 |
|  | Sp2 | 37.8*** | 43.5 | 39.9 | 42.0 | 31.4* | 34.5 |
| Grain protein content (w/w %) | Population | 8.7*** | 10.8 | 12.2*** | 12.8 | 12.8*** | 13.5 |
|  | Sp1 | 8.5*** | 10.8 | 12.0** | 12.7 | 12.7*** | 13.4 |
|  | Sp2 | 9.6** | 11.2 | 13.1 | 13.5 | 13.6 | 14.1 |
| GNACE (kg N in grain/ kg N available) | Population | 1.70*** | 0.52 | 0.21*** | 0.24 | 1.06*** | 0.60 |
|  | Sp1 | 1.71*** | 0.53 | 0.20*** | 0.23 | 1.1*** | 0.62 |
|  | Sp2 | 1.62*** | 0.47 | 0.24 | 0.27 | 0.85*** | 0.51 |

Significant differences between the two treatments were indicated by asterisks as follows:

^*^ p<0.05, ^**^ p<0.01, ^***^p<0.001.

B

| Trait | 2012-2013 | | 2013-2014 | | 2014-2015 | |
| --- | --- | --- | --- | --- | --- | --- |
|  | N0 | N120 | N0 | N120 | N0 | N120 |
| Grain yield (kgha^-1^) | ** | ** | NS | NS | *** | *** |
| Straw yield (kgha^-1^) | NS | NS | NS | * | * | NS |
| TGW (g) | *** | NS | NS | NS | NS | NS |
| NUp_grain_ (kg N in GY) | NS | NS | * | ** | *** | ** |
| NUp_full_ (kg N in biomass) | NS | NS | * | ** | *** | * |
| NUE | ** | ** | NS | NS | *** | *** |
| NUpE | NS | * | * | ** | *** | * |
| NUtE | ** | NS | NS | NS | *** | *** |
| NHI | NS | NS | NS | NS | NS | *** |
| Heading Date (day) | NS | NS | *** | *** | *** | *** |
| Plant height (cm) | NS | NS | NS | NS | NS | NS |
| Spike number per m | *** | ** | NS | NS | ** | NS |
| Grain number per spike | NS | NS | * | ** | *** | *** |
| Harvest-Index | ** | ** | NS | NS | *** | *** |
| Grain protein content (w/w %) | *** | NS | ** | ** | *** | ** |
| GNACE (kg N in grain/ kg N available) | NS | NS | * | ** | *** | ** |

Significant differences between the two subpopulations were indicated by asterisks as follows:

^*^p<0.05, ^**^ p<0.01, ^***^p<0.001

NS: not significant
