# Supplementary material for: Genome-wide association study and genetic diversity analysis on nitrogen use efficiency in a Central European winter wheat (Triticum aestivum L.) collection
Source: PLoS One. 2017 Dec 28;12(12):e0189265. doi: 10.1371/journal.pone.0189265 (PMC5746223; doi:10.1371/journal.pone.0189265)
Supplement: S3 Table — (DOCX) [file pone.0189265.s003.docx]

**S3 Table. Overview of intra-chromosomal LD on each chromosomes in the whole population.**

| **Chromosome** | **No. of marker pairs** | **Mean r^2^ of all marker pairs** | **critical r^2^** | **No of marker pairs in perfect LD** |
| --- | --- | --- | --- | --- |
| 1A | 11,175 | 0.058 | 0.3628 | 31 |
| 2A | 24,531 | 0.070 | 0.3823 | 657 |
| 3A | 10,153 | 0.044 | 0.304 | 21 |
| 4A | 15,051 | 0.081 | 0.3865 | 50 |
| 5A | 10,878 | 0.070 | 0.3089 | 92 |
| 6A | 17,205 | 0.059 | 0.3188 | 61 |
| 7A | 17,391 | 0.048 | 0.2887 | 59 |
| 1B | 58,996 | 0.197 | 0.4371 | 3068 |
| 2B | 40,470 | 0.075 | 0.3905 | 219 |
| 3B | 26,796 | 0.051 | 0.3091 | 86 |
| 4B | 3,741 | 0.068 | 0.351 | 26 |
| 5B | 31,626 | 0.072 | 0.3709 | 248 |
| 6B | 15,931 | 0.072 | 0.3692 | 53 |
| 7B | 31,626 | 0.052 | 0.3405 | 85 |
| 1D | 2,415 | 0.092 | 0.3202 | 85 |
| 2D | 13,041 | 0.294 | 0.3645 | 619 |
| 3D | 2,701 | 0.113 | 0.2788 | 102 |
| 4D | 105 | 0.083 | 0.2137 | 0 |
| 5D | 561 | 0.108 | 0.2862 | 10 |
| 6D | 1,326 | 0.097 | 0.3208 | 21 |
| 7D | 861 | 0.110 | 0.3486 | 18 |

perfect LD: r^2^=1 and D’=1
