# Supplementary material for: Genome-wide association study and genetic diversity analysis on nitrogen use efficiency in a Central European winter wheat (Triticum aestivum L.) collection
Source: PLoS One. 2017 Dec 28;12(12):e0189265. doi: 10.1371/journal.pone.0189265 (PMC5746223; doi:10.1371/journal.pone.0189265)
Supplement: S4 Table — (DOCX) [file pone.0189265.s004.docx]

**S4 Table. Results of analysis of variance for Genotype, Input level, Environment (year) and their Interaction Effects.**

| **Trait** | **Source of variation** | **SS** | **df** | **MS** | **F** | **p** |
| --- | --- | --- | --- | --- | --- | --- |
| Grain yield | G | 85818 | 92 | 903 | 3.4 | 0,000 |
|  | I | 41417 | 1 | 41417 | 156.2 | 0,000 |
|  | E | 292578 | 2 | 146289 | 551.6 | 0,000 |
|  | I xG | 25078 | 92 | 264 | 1.0 | 0,495 |
|  | I xE | 6255 | 2 | 3128 | 11.8 | 0,000 |
|  | G xE | 83423 | 184 | 439 | 1.7 | 0,000 |
| Straw yield | G | 111 | 92 | 1.17 | 2.2 | 0,000 |
|  | I | 9 | 1 | 9.41 | 17.5 | 0,000 |
|  | E | 1288 | 2 | 644 | 1196.1 | 0,000 |
|  | I xG | 54 | 92 | 0,56 | 1.0 | 0,362 |
|  | I xE | 5 | 2 | 2,53 | 4.7 | 0,009 |
|  | G xE | 146 | 184 | 1 | 1.4 | 0,000 |
| Thousand grain weight | G | 18260 | 92 | 192 | 16.5 | 0,000 |
|  | I | 20 | 1 | 20 | 1.7 | 0,190 |
|  | E | 16846 | 2 | 8423 | 723.5 | 0,000 |
|  | I xG | 1073 | 92 | 11 | 1.0 | 0,561 |
|  | I xE | 795 | 2 | 398 | 34.2 | 0,000 |
|  | G xE | 5325 | 184 | 28 | 2.4 | 0,000 |
| NUp_grain_ kg N in yield | G | 164804 | 92 | 1735 | 2.2 | 0,000 |
|  | I | 477778 | 1 | 477778 | 607.5 | 0,000 |
|  | E | 989219 | 2 | 494609 | 628.9 | 0,000 |
|  | I xG | 45215 | 92 | 476 | 0.6 | 0,999 |
|  | I xE | 3178 | 2 | 1589 | 2.0 | 0,133 |
|  | G xE | 254752 | 184 | 1362 | 1.7 | 0,000 |
| NUp_full_ kg N in biomass | G | 208699 | 92 | 2197 | 1.8 | 0,000 |
|  | I | 646676 | 1 | 646676 | 525.6 | 0,000 |
|  | E | 1652272 | 2 | 826136 | 671.4 | 0,000 |
|  | I xG | 74332 | 92 | 782 | 0.6 | 0,997 |
|  | I xE | 5316 | 2 | 2658 | 2.2 | 0,116 |
|  | G xE | 379336 | 184 | 2029 | 1.6 | 0,000 |
| Nitrogen use efficency | G | 40234 | 92 | 424 | 1.5 | 0,003 |
|  | I | 372142 | 1 | 372142 | 1299.6 | 0,000 |
|  | E | 669658 | 2 | 334829 | 1169.3 | 0,000 |
|  | I xG | 19810 | 92 | 209 | 0.7 | 0,974 |
|  | I xE | 402347 | 2 | 201173 | 702.5 | 0,000 |
|  | G xE | 47523 | 184 | 254 | 0.9 | 0,844 |
| Nitrogen uptake  efficency | G | 22 | 92 | 0 | 0.9 | 0,816 |
|  | I | 186 | 1 | 186 | 702,0 | 0,000 |
|  | E | 289 | 2 | 144 | 546.2 | 0,000 |
|  | I xG | 16 | 92 | 0 | 0.6 | 0,998 |
|  | I xE | 159 | 2 | 80 | 301.1 | 0,000 |
|  | G xE | 36 | 184 | 0 | 0.7 | 0,997 |
| Nitrogen utilization  efficiency | G | 3300829 | 92 | 34746 | 2.7 | 0,000 |
|  | I | 2672960 | 1 | 2672960 | 206.5 | 0,000 |
|  | E | 7925219 | 2 | 3962609 | 306.1 | 0,000 |
|  | I xG | 727733 | 92 | 7660 | 0.6 | 0,999 |
|  | I xE | 108059 | 2 | 54030 | 4.2 | 0,016 |
|  | G xE | 2822938 | 184 | 15016 | 1.2 | 0,088 |
| Nitrogen harvest  index | G | 0,74 | 92 | 0.01 | 2.4 | 0,000 |
|  | I | 0,51 | 1 | 0,53 | 165.0 | 0,000 |
|  | E | 1,68 | 2 | 0,82 | 257.6 | 0,000 |
|  | I xG | 0,3 | 92 | 0,082 | 0.9 | 0,721 |
|  | I xE | 0,4 | 2 | 0,00 | 63.8 | 0,000 |
|  | G xE | 1,2 | 184 | 0,20 | 2.1 | 0,000 |
| Heading date | G | 15328 | 92 | 161 | 124.3 | 0,000 |
|  | I | 4 | 1 | 4 | 3.4 | 0,065 |
|  | E | 242920 | 2 | 121460 | 93597.4 | 0,000 |
|  | I xG | 82 | 92 | 1 | 0.7 | 0,994 |
|  | I xE | 25 | 2 | 12 | 9.6 | 0,000 |
|  | G xE | 2152 | 184 | 11 | 8.7 | 0,000 |
| Plant height | G | 76797 | 92 | 808 | 25.3 | 0,000 |
|  | I | 2572 | 1 | 2572 | 80.6 | 0,000 |
|  | E | 500047 | 2 | 250024 | 7836.3 | 0,000 |
|  | I xG | 2547 | 92 | 27 | 0.8 | 0,861 |
|  | I xE | 82 | 2 | 41 | 1.3 | 0,278 |
|  | G xE | 14576 | 184 | 77 | 2.4 | 0,000 |
| Spike number per m | G | 106920 | 92 | 1125 | 5.9 | 0,000 |
|  | I | 7620 | 1 | 7620 | 39.7 | 0,000 |
|  | E | 152349 | 2 | 76175 | 396.6 | 0,000 |
|  | I xG | 20195 | 92 | 213 | 1.1 | 0,233 |
|  | I xE | 92 | 2 | 46 | 0.2 | 0,787 |
|  | G xE | 78796 | 184 | 415 | 2.2 | 0,000 |
| Grain number per spike | G | 100000000 | 92 | 1052887 | 6.7 | 0,000 |
|  | I | 30350000 | 1 | 30350000 | 193.9 | 0,000 |
|  | E | 154600000 | 2 | 77290000 | 493.8 | 0,000 |
|  | I xG | 15840000 | 92 | 166765 | 1.1 | 0,319 |
|  | I xE | 3155266 | 2 | 1577633 | 10.1 | 0,000 |
|  | G xE | 55240000 | 184 | 290735 | 1.9 | 0,000 |
| Harvest index | G | 15291 | 92 | 161 | 7.9 | 0,000 |
|  | I | 3132 | 1 | 3132 | 153.7 | 0,000 |
|  | E | 10191 | 2 | 5095 | 250.0 | 0,000 |
|  | I xG | 1725 | 92 | 18 | 0.9 | 0,761 |
|  | I xE | 1161 | 2 | 581 | 28.5 | 0,000 |
|  | G xE | 8877 | 184 | 47 | 2.3 | 0,000 |
| Grain protein  content | G | 889 | 92 | 9 | 4.3 | 0,000 |
|  | I | 399 | 1 | 399 | 183.1 | 0,000 |
|  | E | 2636 | 2 | 1318 | 604.4 | 0,000 |
|  | I xG | 164 | 92 | 2 | 0.8 | 0,926 |
|  | I xE | 173 | 2 | 86 | 39.6 | 0,000 |
|  | G xE | 475 | 184 | 3 | 1.2 | 0,081 |
| GNACE | G | 13 | 92 | 0 | 0.9 | 0,642 |
|  | I | 86 | 1 | 86 | 585.8 | 0,000 |
|  | E | 151 | 2 | 75 | 514.7 | 0,000 |
|  | I xG | 8 | 92 | 0 | 0.6 | 0,999 |
|  | I xE | 77 | 2 | 39 | 263.3 | 0,000 |
|  | G xE | 20 | 184 | 0 | 0.7 | 0,997 |

G:Genotype; I:Input level; E:Environment
