# Supplementary material for: Genome-wide association study and genetic diversity analysis on nitrogen use efficiency in a Central European winter wheat (Triticum aestivum L.) collection
Source: PLoS One. 2017 Dec 28;12(12):e0189265. doi: 10.1371/journal.pone.0189265 (PMC5746223; doi:10.1371/journal.pone.0189265)
Supplement: S5 Table — (DOCX) [file pone.0189265.s005.docx]

**S5 Table. Overview of markers associated with multiple traits.**

| **Trait** | **Year** | **Chr** | **Position (cM)** | **Marker name** | **N input level** |
| --- | --- | --- | --- | --- | --- |
| NUE | 2013 | 1A | 15 | 1143089 | 120 |
| NUpfull | 2013 | 1A | 15 | 1143089 | 120 |
| NUpE | 2014 | 1B | 8 | 1089620 | 0 |
| NUpfull | 2014 | 1B | 8 | 1089620 | 0 |
| NUpfull_RN | 2014 | 1B | 8 | 1089620 |  |
| GNACE | 2014 | 1B | 18 | 1103430 | 0 |
| NUpE | 2014 | 1B | 18 | 1103430 | 0 |
| NUpgrain | 2014 | 1B | 18 | 1103430 | 0 |
| NUpfull | 2014 | 1B | 18 | 1103430 | 0 |
| GNACE | 2014 | 1B | 130 | 1724416 | 0 |
| NUpE | 2014 | 1B | 130 | 1724416 | 0 |
| NUpgrain | 2014 | 1B | 130 | 1724416 | 0 |
| NUpfull | 2014 | 1B | 130 | 1724416 | 0 |
| NUpE | 2014 | 1B | 147 | 1235415 | 0 |
| NUpgrain | 2014 | 1B | 147 | 1235415 | 0 |
| NUpfull | 2014 | 1B | 147 | 1235415 | 0 |
| GNACE | 2013 | 2B | 1 | 1035639 | 0 |
| NUpgrain | 2013 | 2B | 1 | 1035639 | 0 |
| GNACE | 2015 | 2B | 25 | 1120906 | 120 |
| NUE | 2015 | 2B | 25 | 1120906 | 120 |
| NUpgrain | 2015 | 2B | 25 | 1120906 | 120 |
| GNACE | 2013 | 2B | 198 | 1245384 | 120 |
| NUpE | 2013 | 2B | 198 | 1245384 | 120 |
| NUpgrain | 2013 | 2B | 198 | 1245384 | 120 |
| NUpfull | 2013 | 2B | 198 | 1245384 | 120 |
| NUpfull_RN | 2015 | 2D | 139 | 991221 |  |
| NUpgrain_RN | 2015 | 2D | 139 | 991221 |  |
| GNACE | 2015 | 2D | 249 | 3385285 | 0 |
| NUpgrain | 2015 | 2D | 249 | 3385285 | 0 |
| NUE | 2014 | 3A | 41 | 1117907 | 0 |
| NUpE | 2014 | 3A | 41 | 1117907 | 0 |
| NUpgrain | 2014 | 3A | 41 | 1117907 | 0 |
| NUpfull | 2014 | 3A | 41 | 1117907 | 0 |
| NHI | 2014 | 3B | 172 | 1104615 | 0 |
| NUpgrain | 2014 | 3B | 172 | 1104615 | 120 |
| GY_RN | 2014 | 3B | 261 | 994423 |  |
| NUpgrain_RN | 2014 | 3B | 261 | 994423 |  |
| GNACE | 2014 | 3B | 298 | 1113201 | 0 |
| NUpfull | 2014 | 3B | 298 | 1113201 | 0 |
| GPC | 2014 | 4A | 249 | 1040398 | 0 |
| NUtE | 2014 | 4A | 249 | 1040398 | 120 |
| GNACE | 2014 | 4B | 91 | 1024824 | 120 |
| NUE | 2014 | 4B | 91 | 1024824 | 120 |
| NUpgrain | 2014 | 4B | 91 | 1024824 | 120 |
| GNACE | 2014 | 5A | 97 | 2259167 | 0 |
| NUpgrain | 2014 | 5A | 97 | 2259167 | 0 |
| GY | 2013 | 5A | 100 | 1087592 | 120 |
| GPC | 2015 | 5A | 100 | 1087592 | 0 |
| GNACE | 2014 | 5A | 294 | 1111119 | 0 |
| NUpE | 2014 | 5A | 294 | 1111119 | 0 |
| NUpgrain | 2014 | 5A | 294 | 1111119 | 0 |
| NUpfull | 2014 | 5A | 294 | 1111119 | 0 |
| GNACE | 2014 | 5B | 47 | 1104310 | 120 |
| NUpgrain | 2014 | 5B | 47 | 1104310 | 120 |
| SN | 2015 | 5B | 93 | 1020159 | 0 |
| GNACE | 2015 | 5B | 93 | 1020159 | 0 |
| NUpE | 2015 | 5B | 93 | 1020159 | 0 |
| NUpgrain | 2015 | 5B | 93 | 1020159 | 0 |
| NUpfull | 2015 | 5B | 93 | 1020159 | 0 |
| NUpE | 2014 | 5B | 148 | 1090414 | 120 |
| NUpfull | 2014 | 5B | 148 | 1090414 | 120 |
| NUtE | 2015 | 5B | 284 | 1139663 | 120 |
| GN_RN | 2014 | 5B | 284 | 1139663 |  |
| GPC | 2014 | 6B | 87 | 1121033 | 120 |
| GPC | 2015 | 6B | 87 | 1121033 | 120 |
| NUtE | 2014 | 6B | 87 | 1121033 | 120 |
| NHI_RN | 2015 | 7A | 32 | 1060475 |  |
| NUtE_RN | 2015 | 7A | 32 | 1060475 |  |
| GN_RN | 2015 | 7A | 196 | 1008272 |  |
| GY_RN | 2015 | 7A | 196 | 1008272 |  |
| GPC_RN | 2013 | 7A | 260 | 3026780 |  |
| NUpfull_RN | 2013 | 7A | 260 | 3026780 |  |
| NUtE_RN | 2013 | 7A | 260 | 3026780 |  |
| GNACE | 2015 | 7A | 286 | 1107195 | 120 |
| NUE | 2015 | 7A | 286 | 1107195 | 120 |
| NUpE | 2015 | 7A | 286 | 1107195 | 120 |
| NUpgrain | 2015 | 7A | 286 | 1107195 | 120 |
| NUpfull | 2015 | 7A | 286 | 1107195 | 120 |
