# Supplementary figures and images for: Genome-wide association study and genetic diversity analysis on nitrogen use efficiency in a Central European winter wheat (Triticum aestivum L.) collection
Source: PLoS One. 2017 Dec 28;12(12):e0189265. doi: 10.1371/journal.pone.0189265 (PMC5746223; doi:10.1371/journal.pone.0189265)

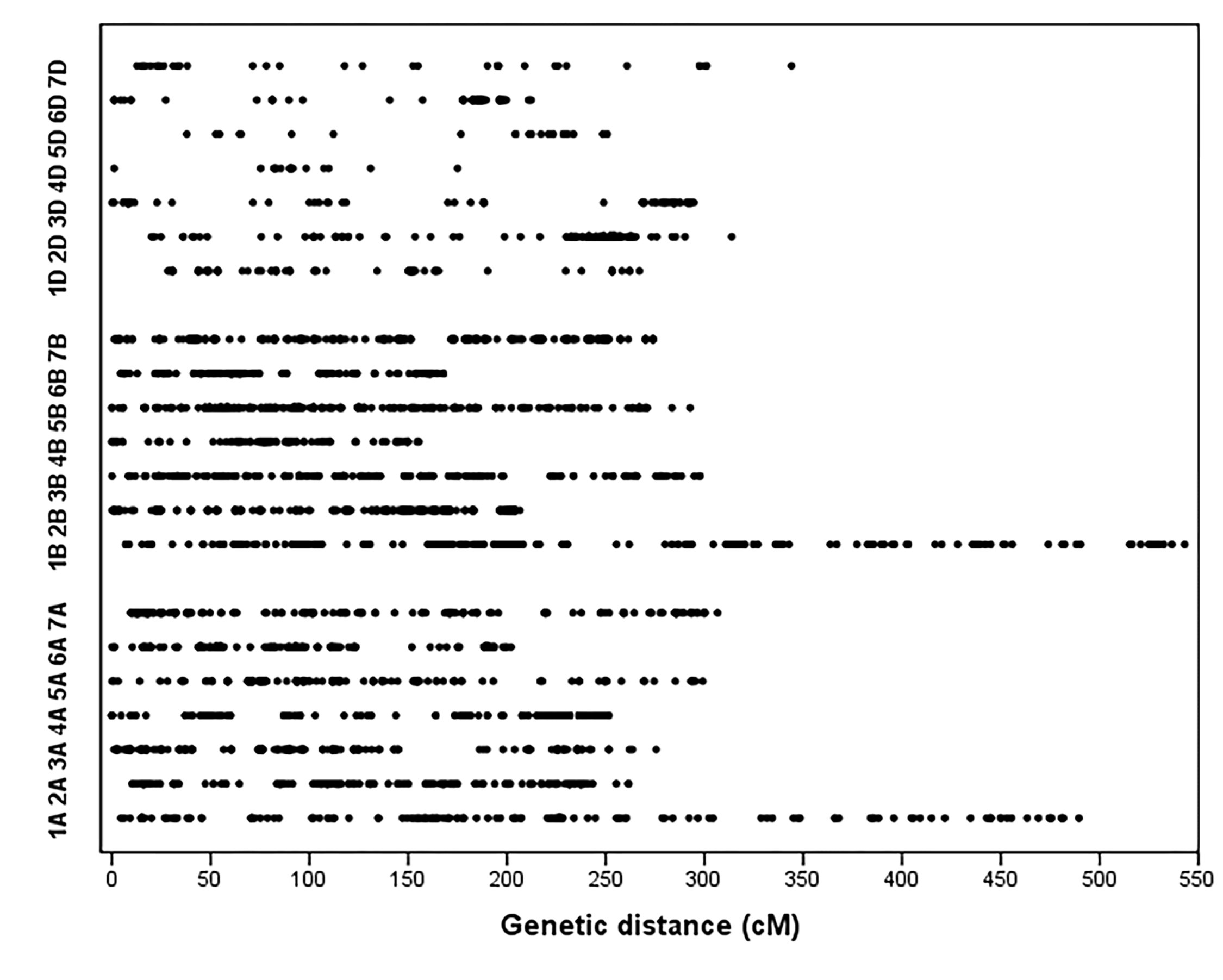

Supplement: S1 Fig — Map information was provided by Triticarte Pty. Ltd. (TIF) [file pone.0189265.s006.tif]
