# Supplementary material for: Genome-wide association study and genetic diversity analysis on nitrogen use efficiency in a Central European winter wheat (Triticum aestivum L.) collection
Source: PLoS One. 2017 Dec 28;12(12):e0189265. doi: 10.1371/journal.pone.0189265 (PMC5746223; doi:10.1371/journal.pone.0189265)

**S2 Fig. Phylogram of 93 winter wheat genotypes showing the phylogenetic relationships of the two subpopulations (bootstrap values also shown).**

Sp 2

Varieties belonging to subpopulation 2 (Sp2) are indicated by the bracket.
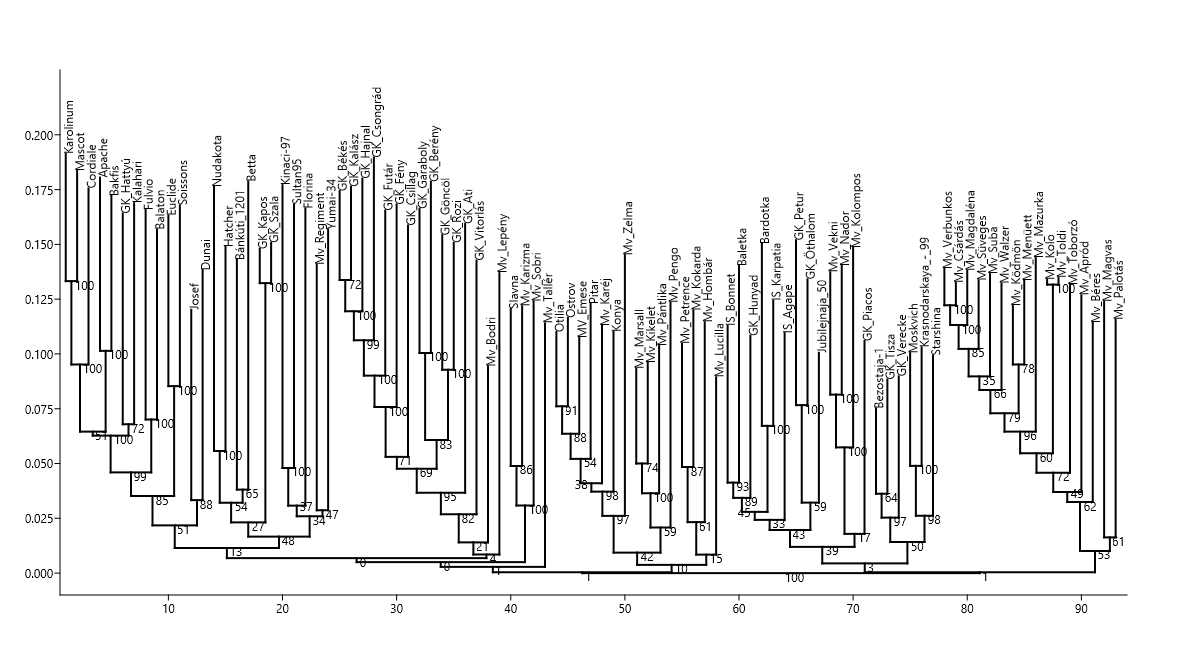

Supplement: S2 Fig — (DOCX) [file pone.0189265.s007.docx]
