# Supplementary material for: Genome-wide association study and genetic diversity analysis on nitrogen use efficiency in a Central European winter wheat (Triticum aestivum L.) collection
Source: PLoS One. 2017 Dec 28;12(12):e0189265. doi: 10.1371/journal.pone.0189265 (PMC5746223; doi:10.1371/journal.pone.0189265)

**S3 Fig. Quantile-Quantile (Q-Q) plot of marker-trait associations for grain yield in three cropping years in N0 treatments, based on MLM-K+Q data.**


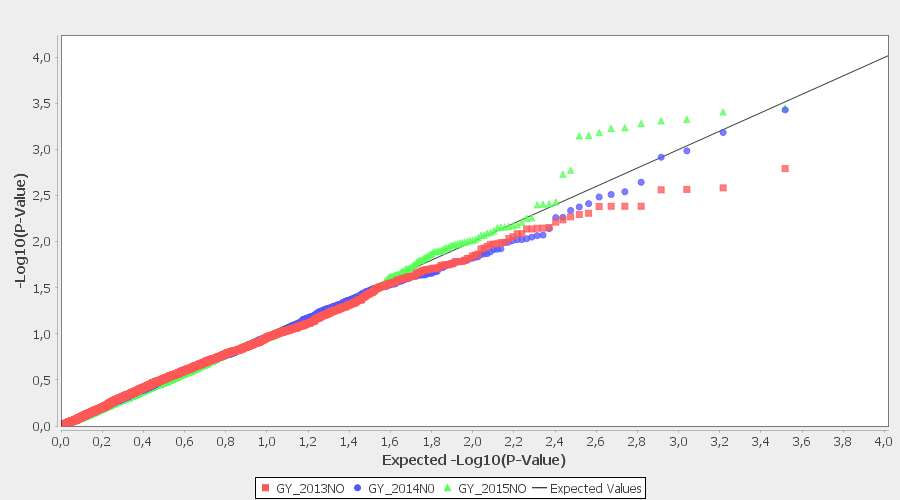


**S2 Fig. Quantile-Quantile (Q-Q) plot of marker-trait associations for grain yield in three cropping years in N120 treatments, based on MLM-K+Q data.**


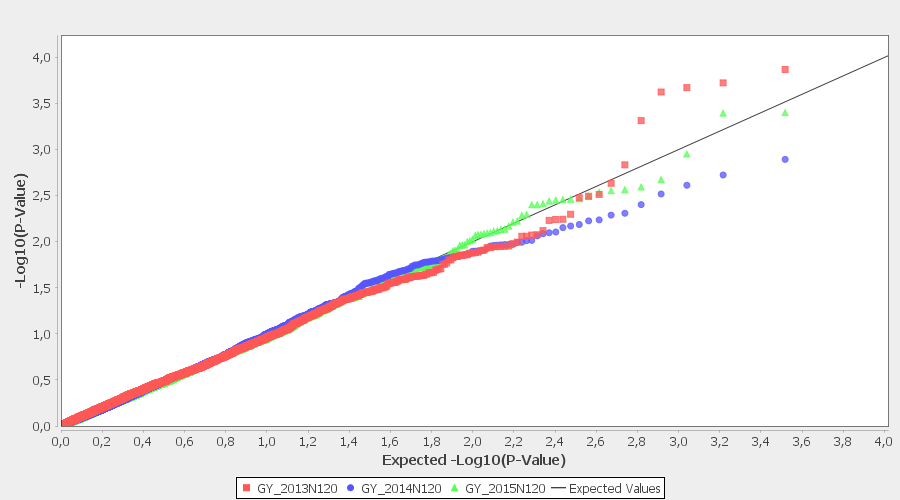


**S2 Fig. Quantile-Quantile (Q-Q) plot of marker-trait associations for grain number per spike in three cropping years in N0 treatments, based on MLM-K+Q data.**


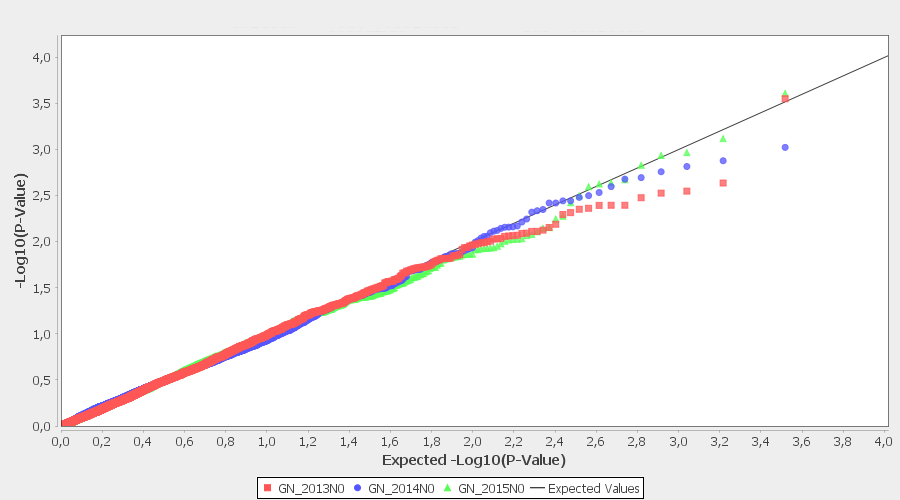


**S2 Fig. Quantile-Quantile (Q-Q) plot of marker-trait associations for grain number per spike in three cropping years in N120 treatments, based on MLM-K+Q data.**


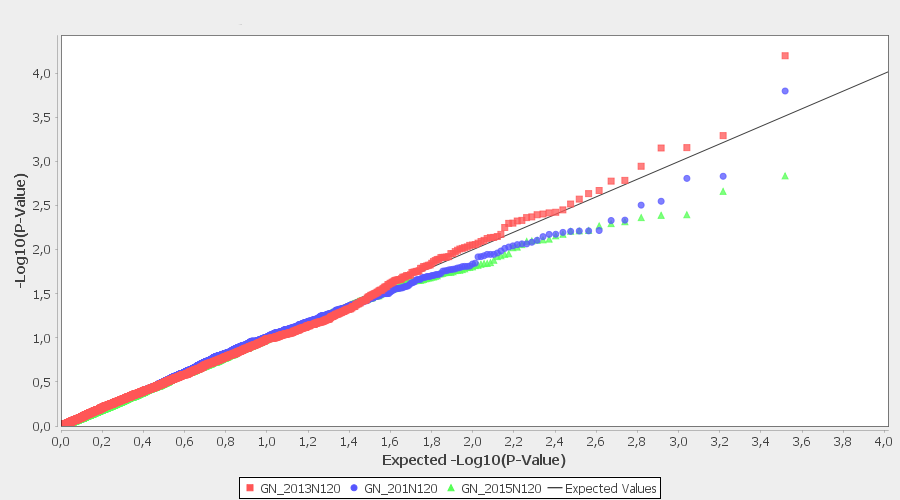


**S2 Fig. Quantile-Quantile (Q-Q) plot of marker-trait associations for spike number per meter in three cropping years in N0 treatments, based on MLM-K+Q data.**


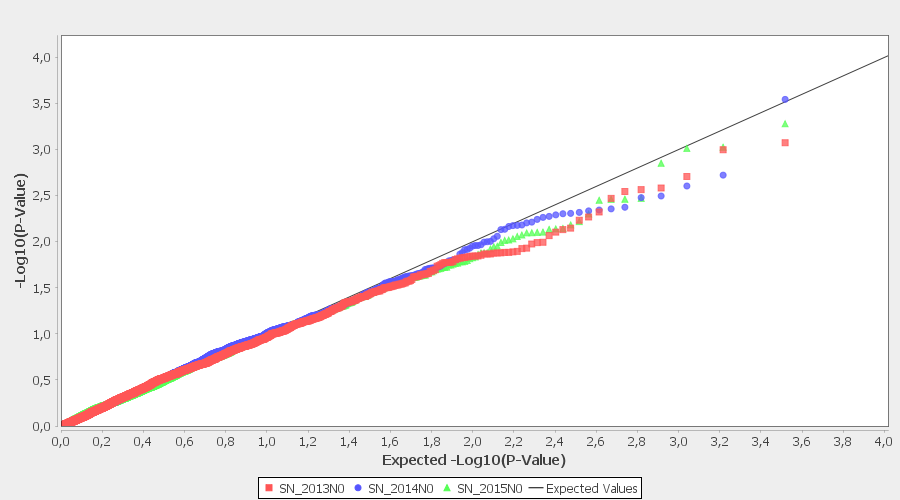


**S2 Fig. Quantile-Quantile (Q-Q) plot of marker-trait associations for spike number per meter in three cropping years in N120 treatments, based on MLM-K+Q data.**


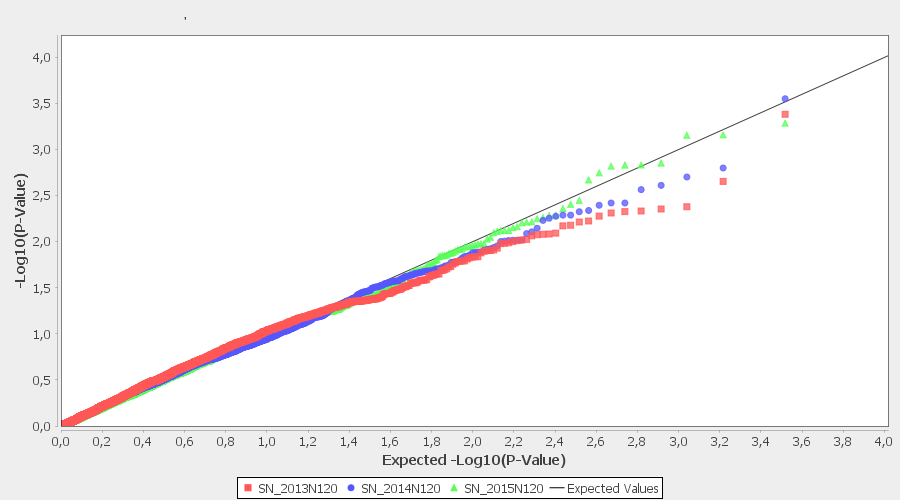


**S2 Fig. Quantile-Quantile (Q-Q) plot of marker-trait associations for nitrogen use efficiency in three cropping years in N0 treatments, based on MLM-K+Q data.**


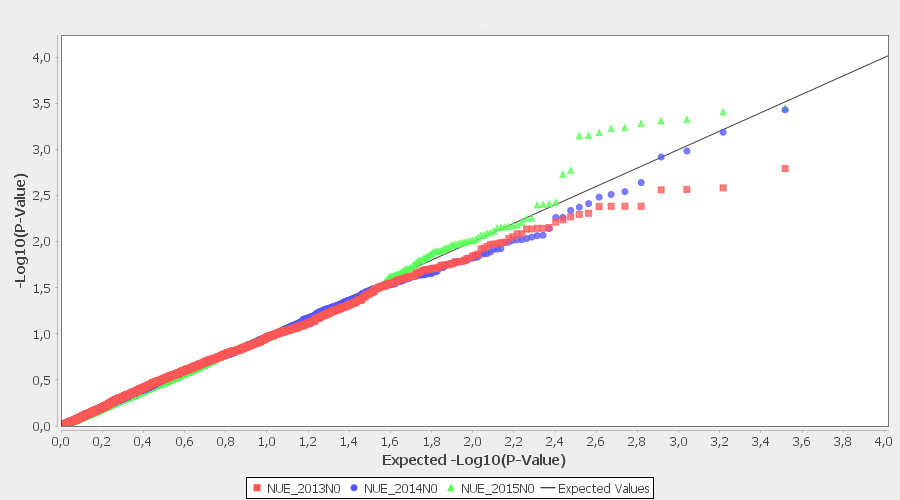


**S2 Fig. Quantile-Quantile (Q-Q) plot of marker-trait associations for nitrogen use efficiency in three cropping years in N120 treatments, based on MLM-K+Q data.**


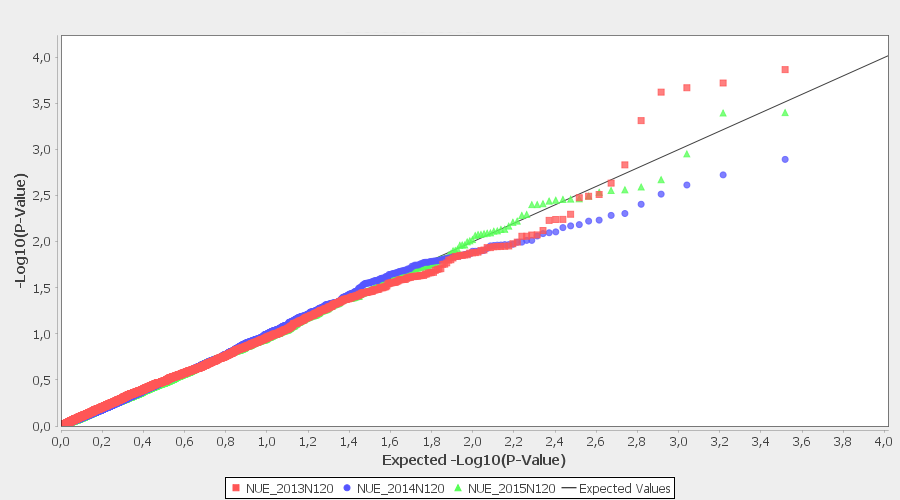


**S2 Fig. Quantile-Quantile (Q-Q) plot of marker-trait associations for nitrogen uptake efficiency in three cropping years in N0 treatments, based on MLM-K+Q data.**


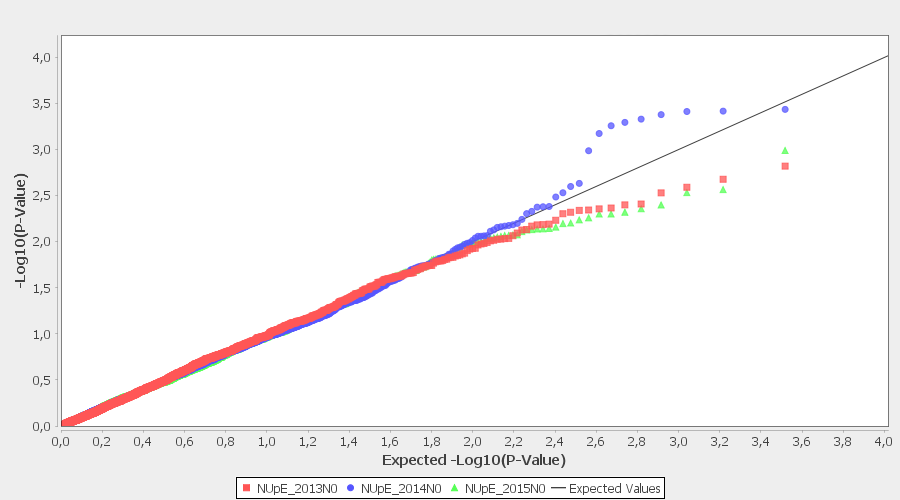


**S2 Fig. Quantile-Quantile (Q-Q) plot of marker-trait associations for nitrogen uptake efficiency in three cropping years in N120 treatments, based on MLM-K+Q data.**


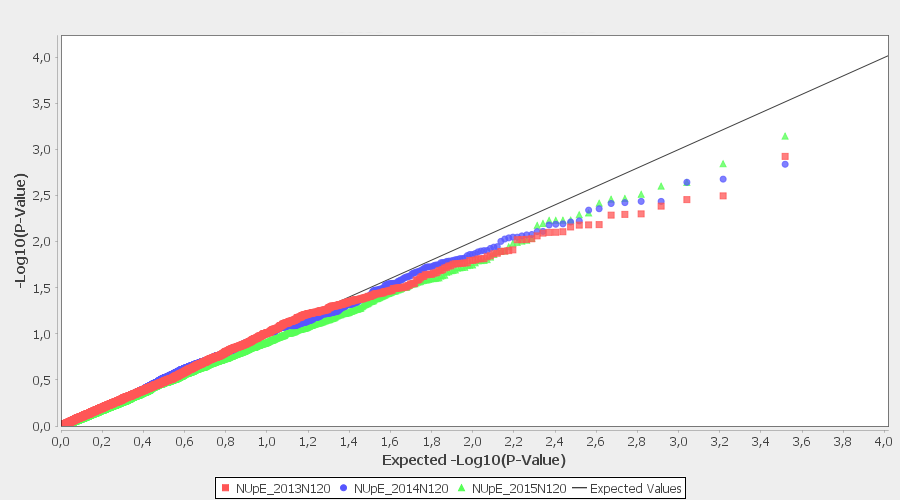


**S2 Fig. Quantile-Quantile (Q-Q) plot of marker-trait associations for nitrogen utilization efficiency in three cropping years in N0 treatments, based on MLM-K+Q data.**


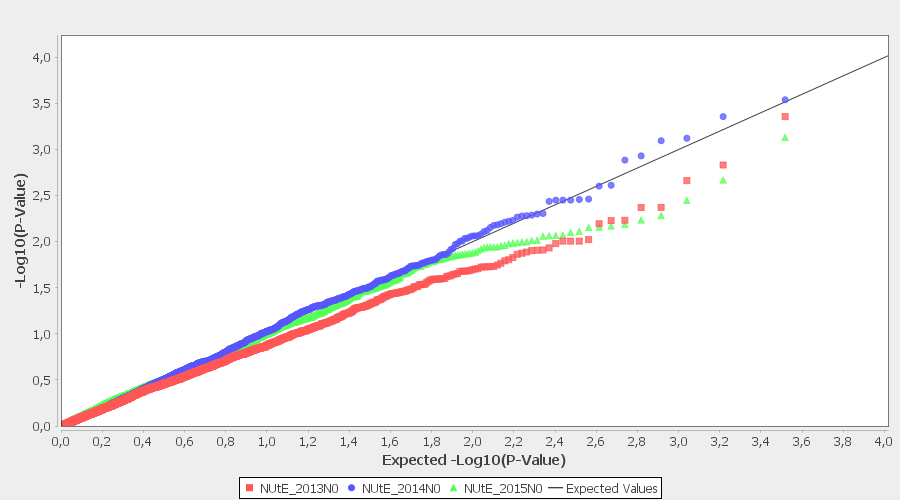


**S2 Fig. Quantile-Quantile (Q-Q) plot of marker-trait associations for nitrogen utilization efficiency in three cropping years in N120 treatments, based on MLM-K+Q data.**


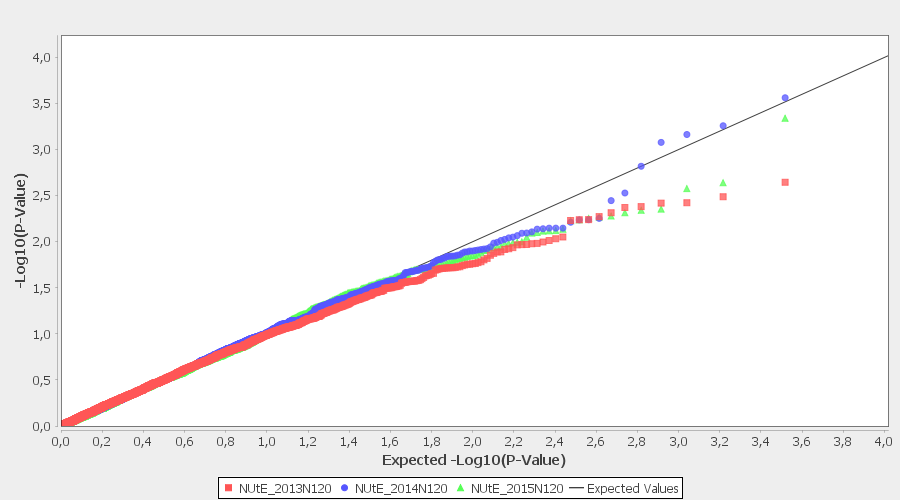


**S2 Fig. Quantile-Quantile (Q-Q) plot of marker-trait associations for the total nitrogen content harvested in the grain in three cropping years in N0 treatments, based on MLM-K+Q data.**


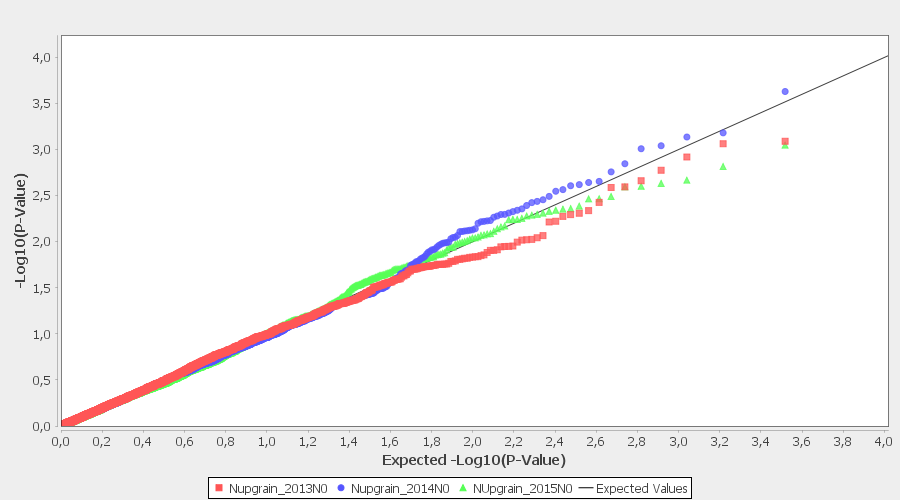


**S2 Fig. Quantile-Quantile (Q-Q) plot of marker-trait associations for the total nitrogen content harvested in the grain in three cropping years in N120 treatments, based on MLM-K+Q data.**


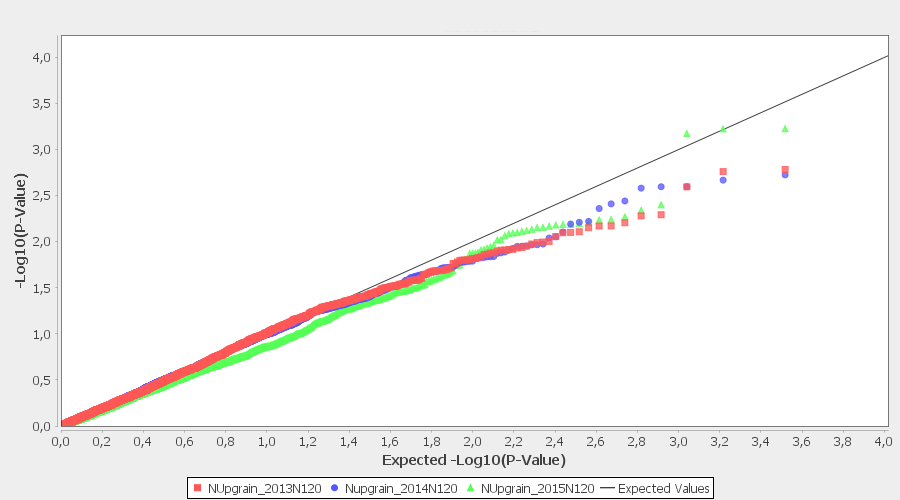


**S2 Fig. Quantile-Quantile (Q-Q) plot of marker-trait associations for grain nitrogen accumulation efficiency in three cropping years in N0 treatments, based on MLM-K+Q data.**


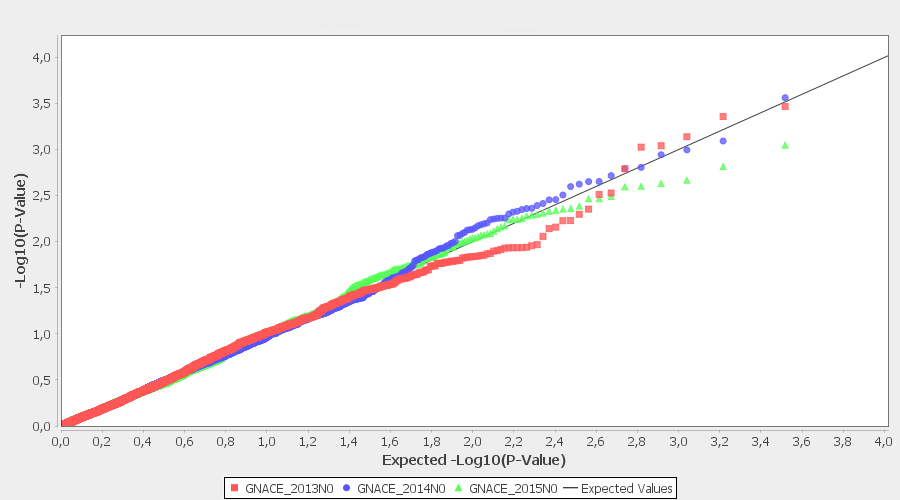


**S2 Fig. Quantile-Quantile (Q-Q) plot of marker-trait associations for grain nitrogen accumulation efficiency in three cropping years in N120 treatments, based on MLM-K+Q data.**


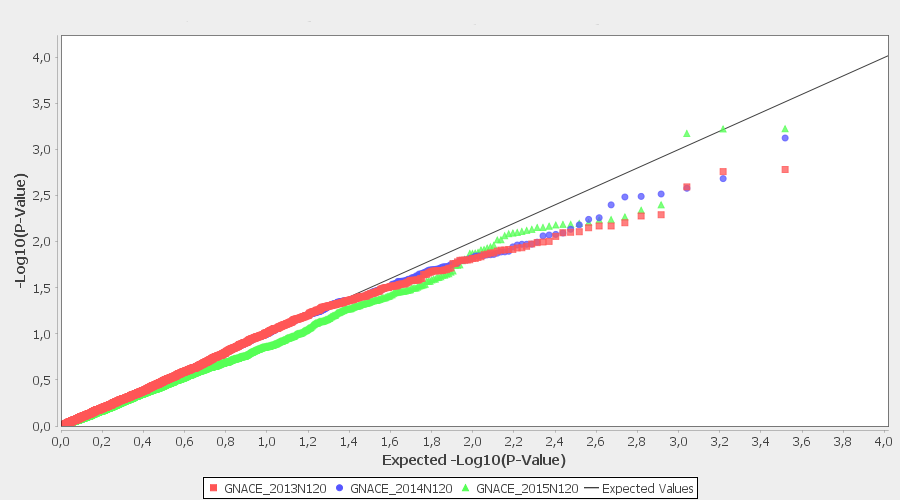


**S2 Fig. Quantile-Quantile (Q-Q) plot of marker-trait associations for the amount of nitrogen taken up by the whole aboveground plant in three cropping years in N0 treatments, based on MLM-K+Q data.**


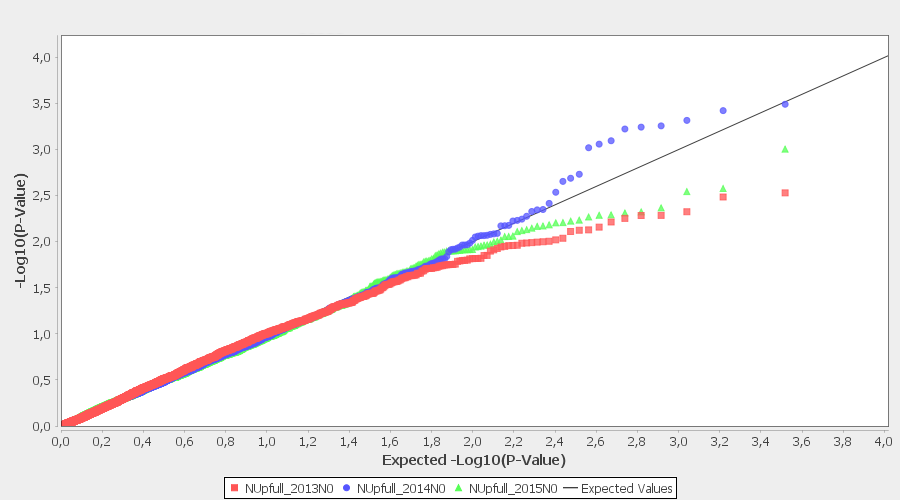


**S2 Fig. Quantile-Quantile (Q-Q) plot of marker-trait associations for the amount of nitrogen taken up by the whole aboveground plant in three cropping years in N120 treatments, based on MLM-K+Q data.**


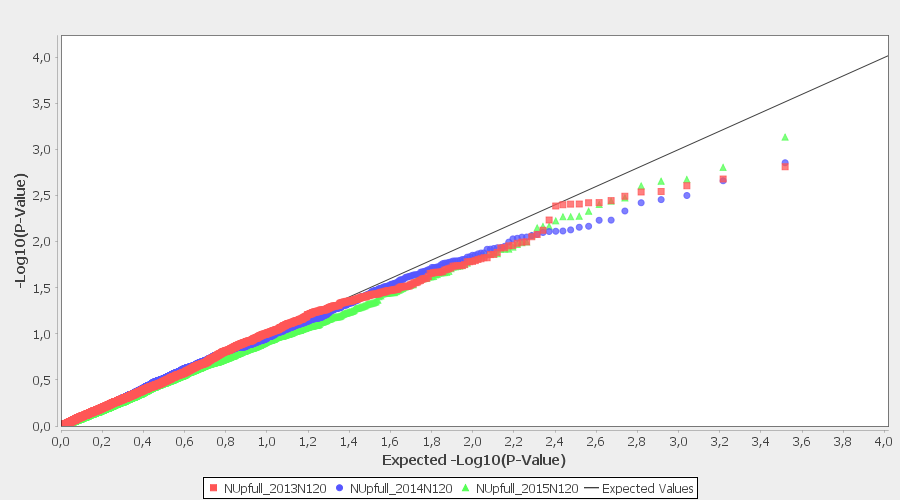


**S2 Fig. Quantile-Quantile (Q-Q) plot of marker-trait associations for nitrogen harvest index in three cropping years in N0 treatments, based on MLM-K+Q data.**


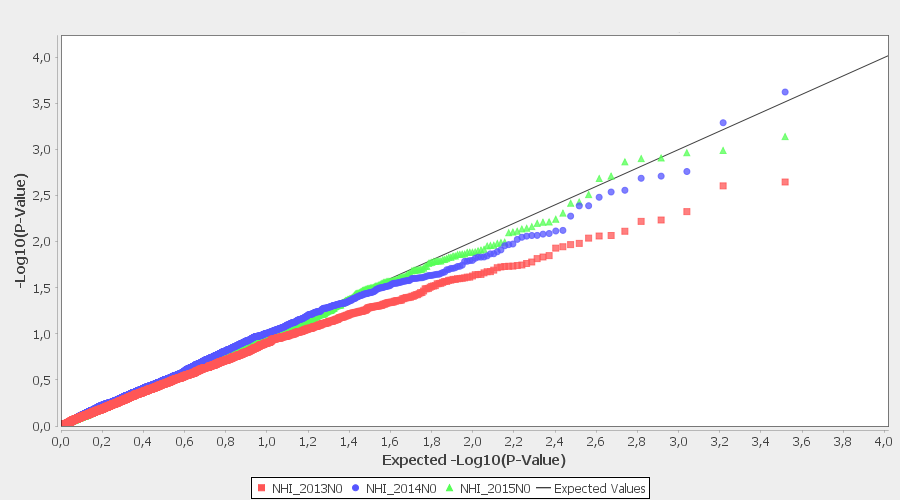


**S2 Fig. Quantile-Quantile (Q-Q) plot of marker-trait associations for nitrogen harvest index in three cropping years in N120 treatments, based on MLM-K+Q data.**


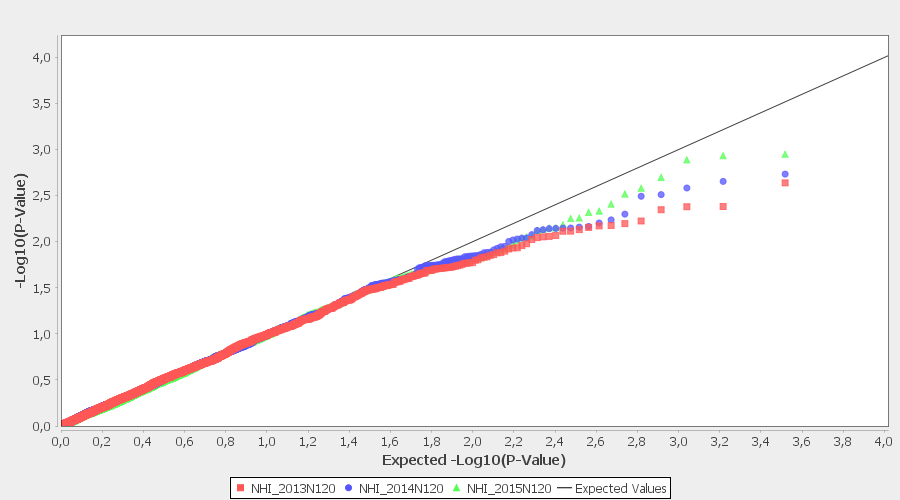


**S2 Fig. Quantile-Quantile (Q-Q) plot of marker-trait associations for grain protein content in three cropping years in N0 treatments, based on MLM-K+Q data.**


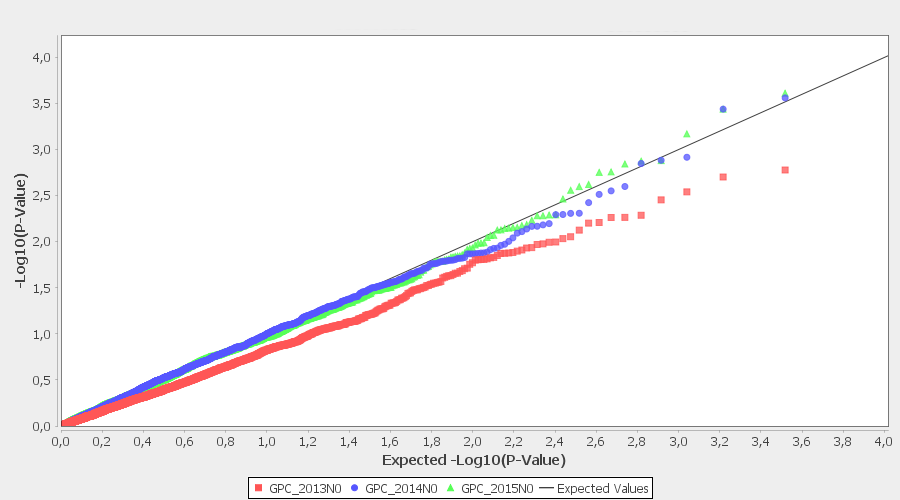


**S2 Fig. Quantile-Quantile (Q-Q) plot of marker-trait associations for grain protein content in three cropping years in N120 treatments, based on MLM-K+Q data.**


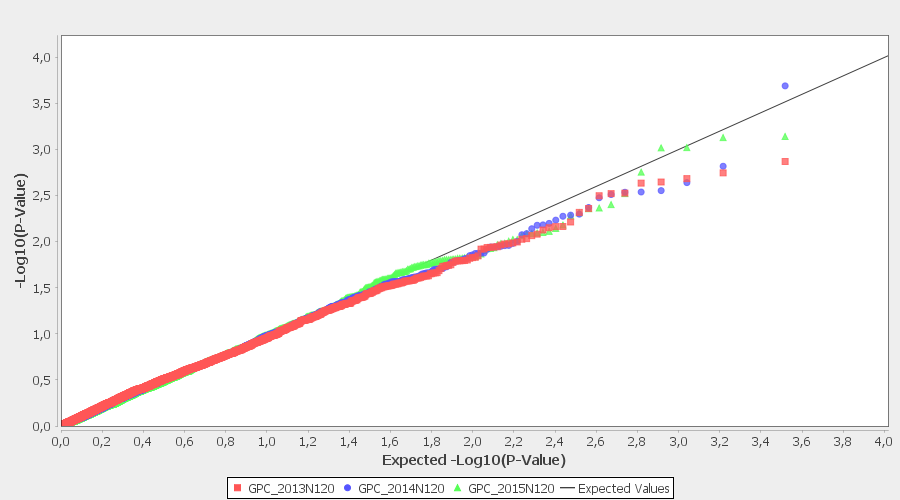


**S2 Fig. Quantile-Quantile (Q-Q) plot of marker-trait associations for grain protein content response to nitrogen fertilization in three cropping years, based on MLM-K+Q data.**


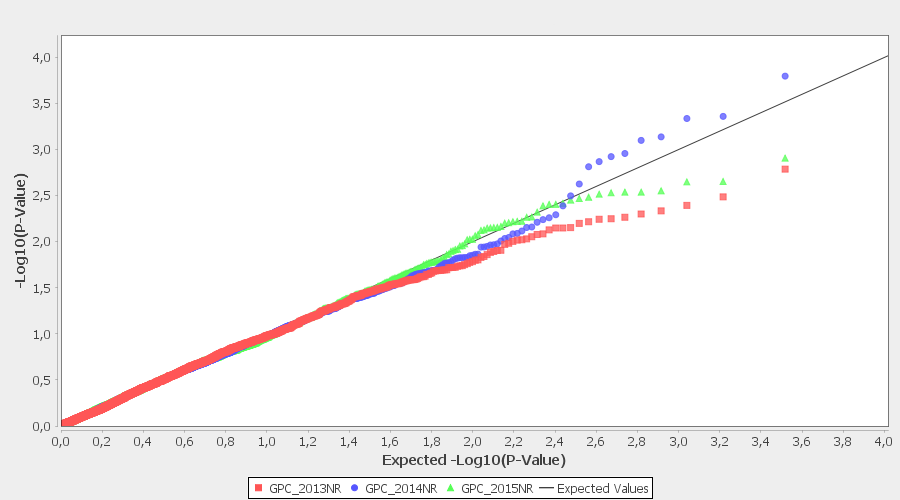


**S2 Fig. Quantile-Quantile (Q-Q) plot of marker-trait associations for nitrogen utilization efficiency response to nitrogen fertilization in three cropping years, based on MLM-K+Q data.**


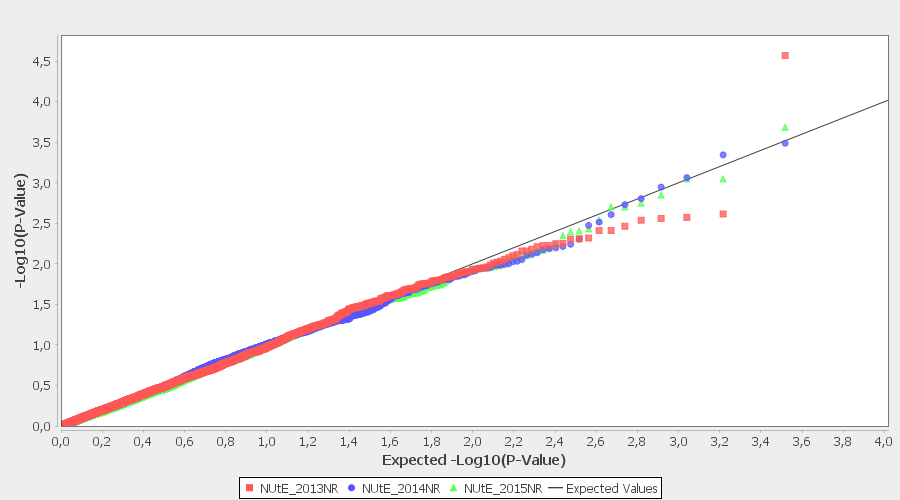


**S2 Fig. Quantile-Quantile (Q-Q) plot of marker-trait associations for nitrogen harvest index response to nitrogen fertilization in three cropping years, based on MLM-K+Q data.**


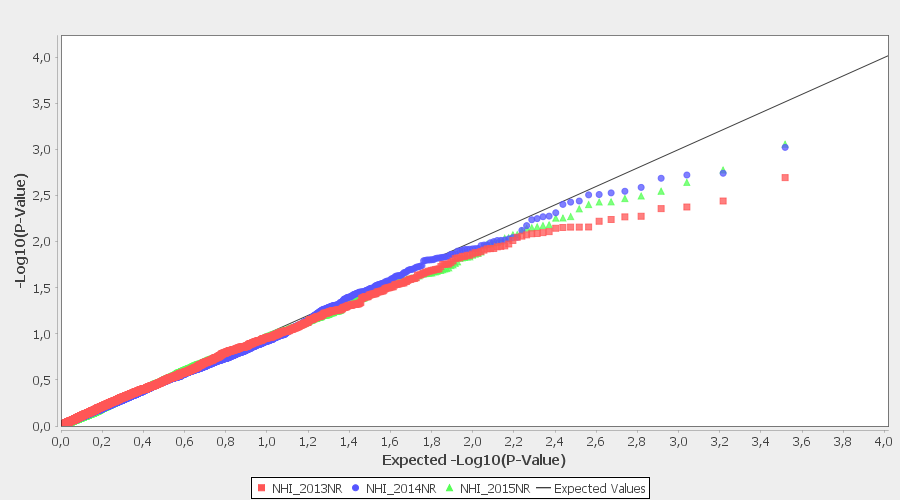


**S2 Fig. Quantile-Quantile (Q-Q) plot of marker-trait associations for total nitrogen content harvested in the grain response to nitrogen fertilization in three cropping years, based on MLM-K+Q data.**


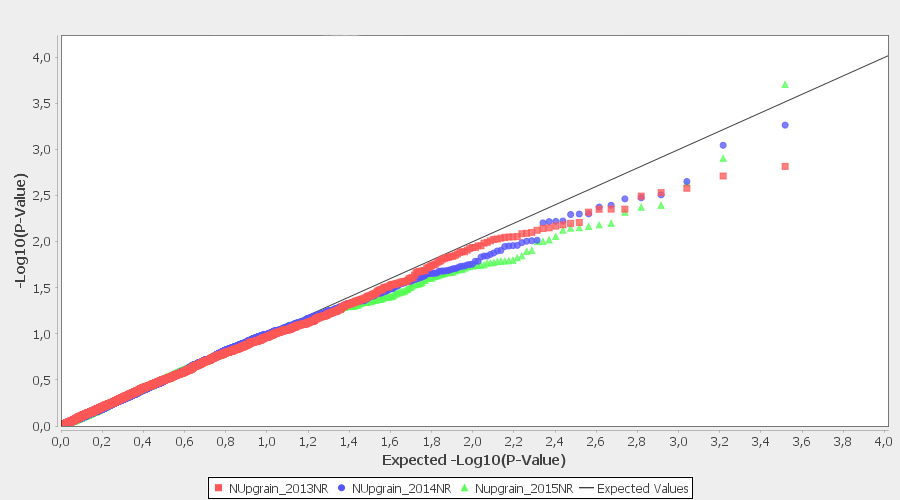


**S2 Fig. Quantile-Quantile (Q-Q) plot of marker-trait associations for** **amount of nitrogen taken up by the whole aboveground plant response to nitrogen fertilization in three cropping years, based on MLM-K+Q data.**


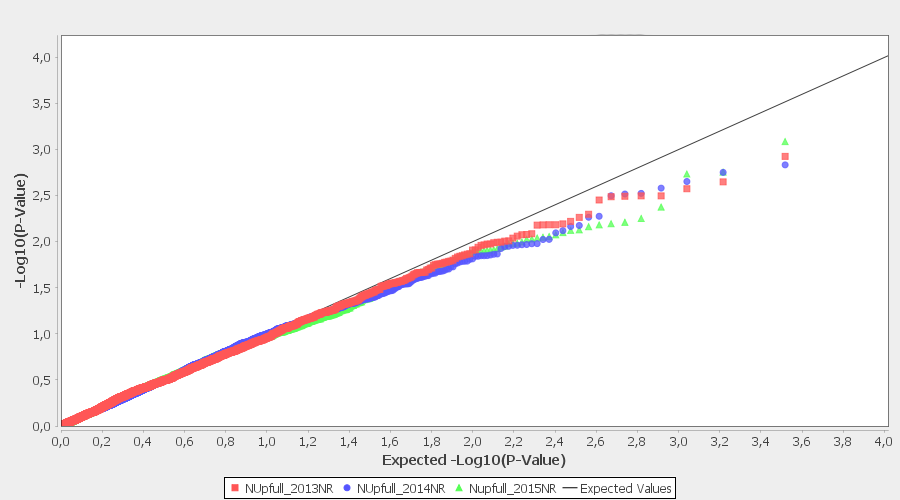


**S2 Fig. Quantile-Quantile (Q-Q) plot of marker-trait associations for** **grain yield response to N fertilization in three cropping years, based on MLM-K+Q data.**


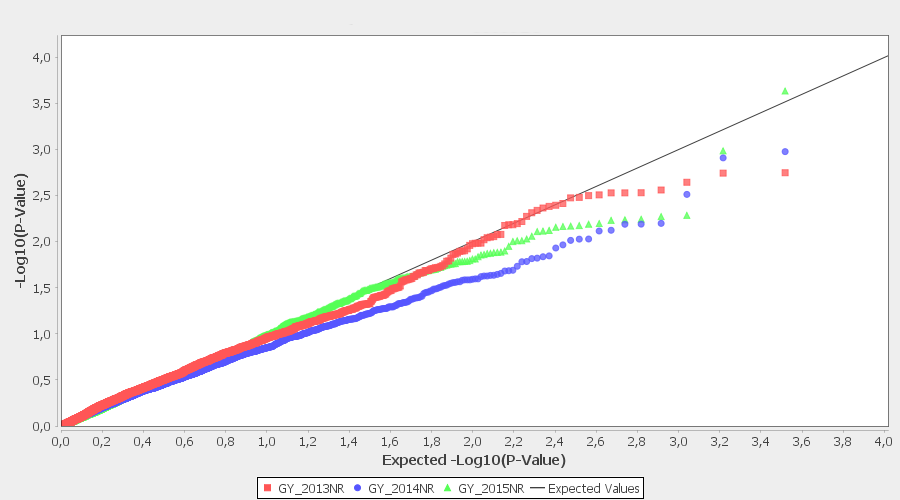


**S2 Fig. Quantile-Quantile (Q-Q) plot of marker-trait associations for** **grain number per spike response to nitrogen fertilization in three cropping years, based on MLM-K+Q data.**


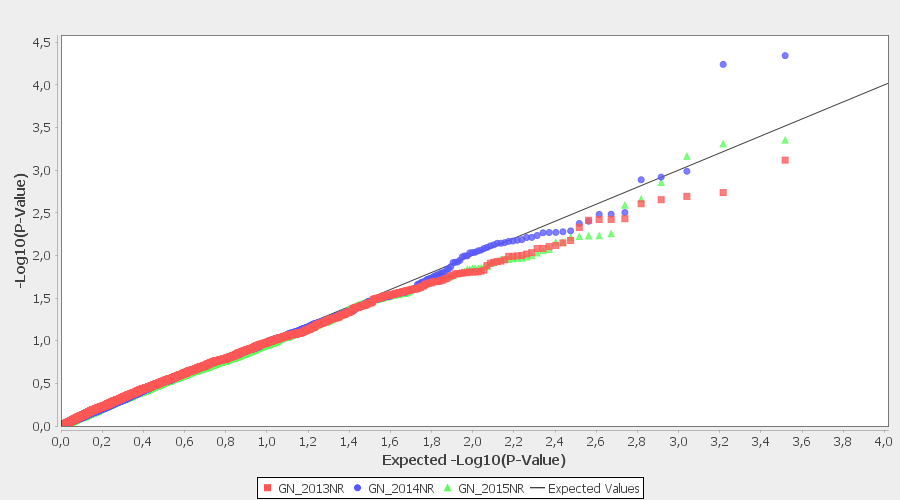


**S2 Fig. Quantile-Quantile (Q-Q) plot of marker-trait associations for** s**pike number per meter response to nitrogen fertilization in three cropping years, based on MLM-K+Q data.**


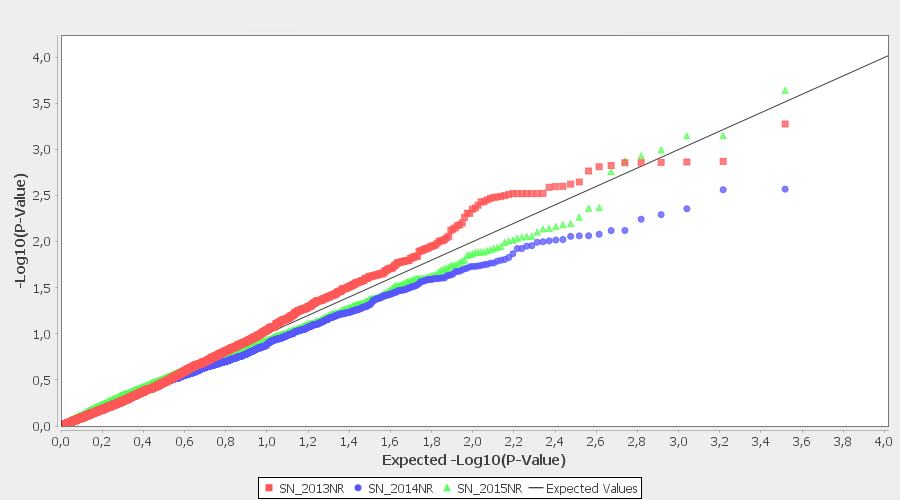

Supplement: S3 Fig — (DOCX) [file pone.0189265.s008.docx]
