# Supplementary material for: Genome-wide association study and genetic diversity analysis on nitrogen use efficiency in a Central European winter wheat (Triticum aestivum L.) collection
Source: PLoS One. 2017 Dec 28;12(12):e0189265. doi: 10.1371/journal.pone.0189265 (PMC5746223; doi:10.1371/journal.pone.0189265)

**S4 Fig. Graphs showing the phenotypic distribution of NUE in 2013 in extensive management.**


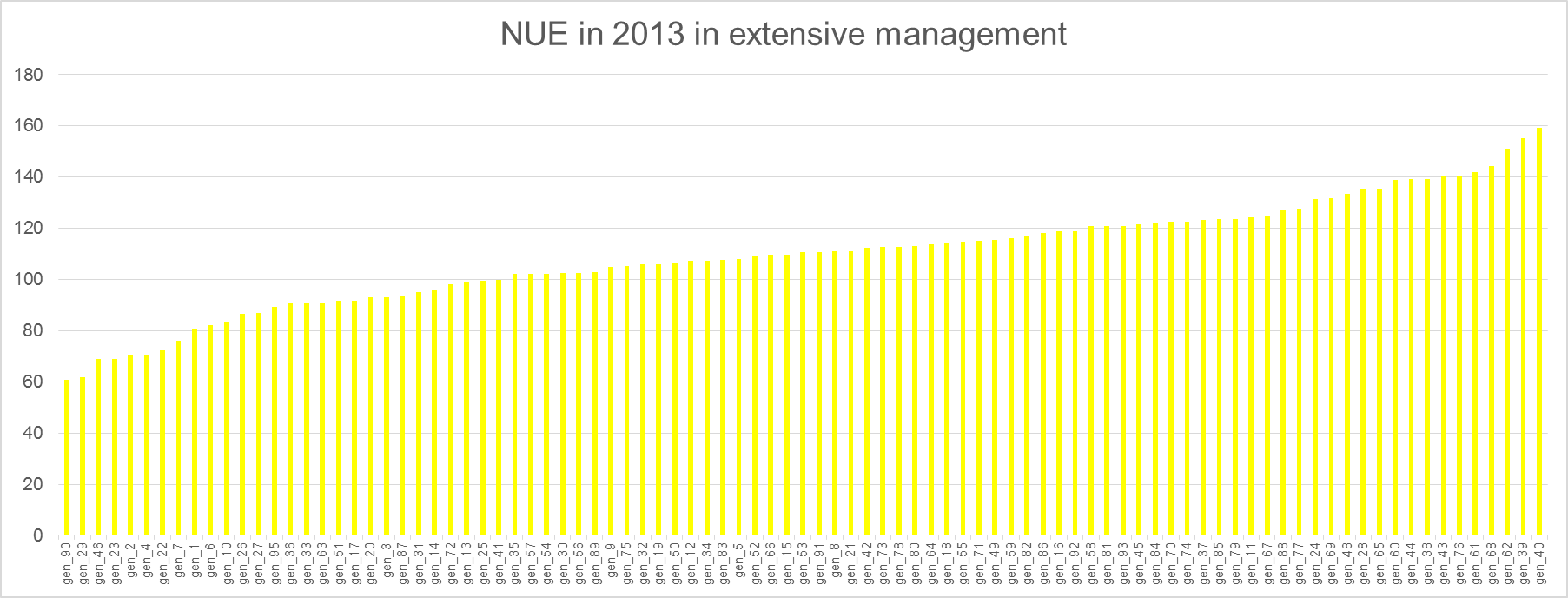


**S4 Fig. Graphs showing the phenotypic distribution of NUE in 2013 in intensive management.**


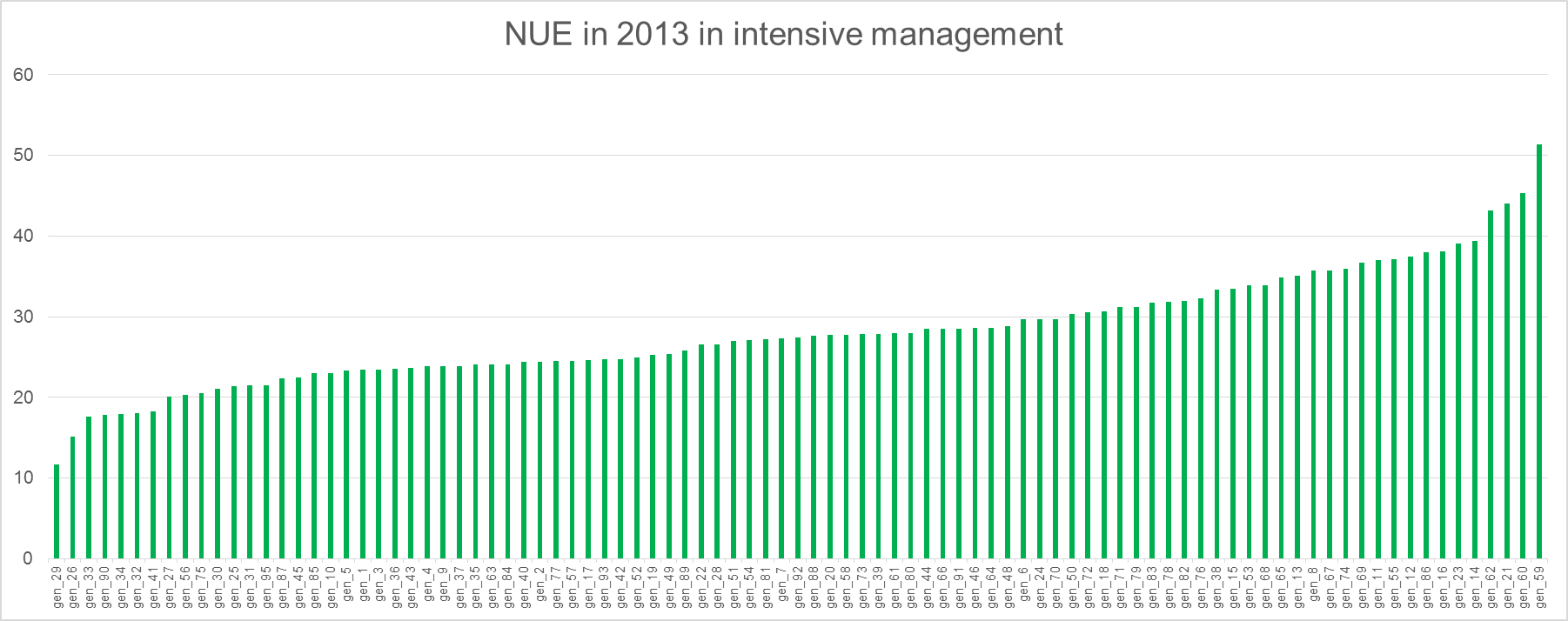


**S4 Fig. Graphs showing the phenotypic distribution of NUE in 2014 in extensive management.**


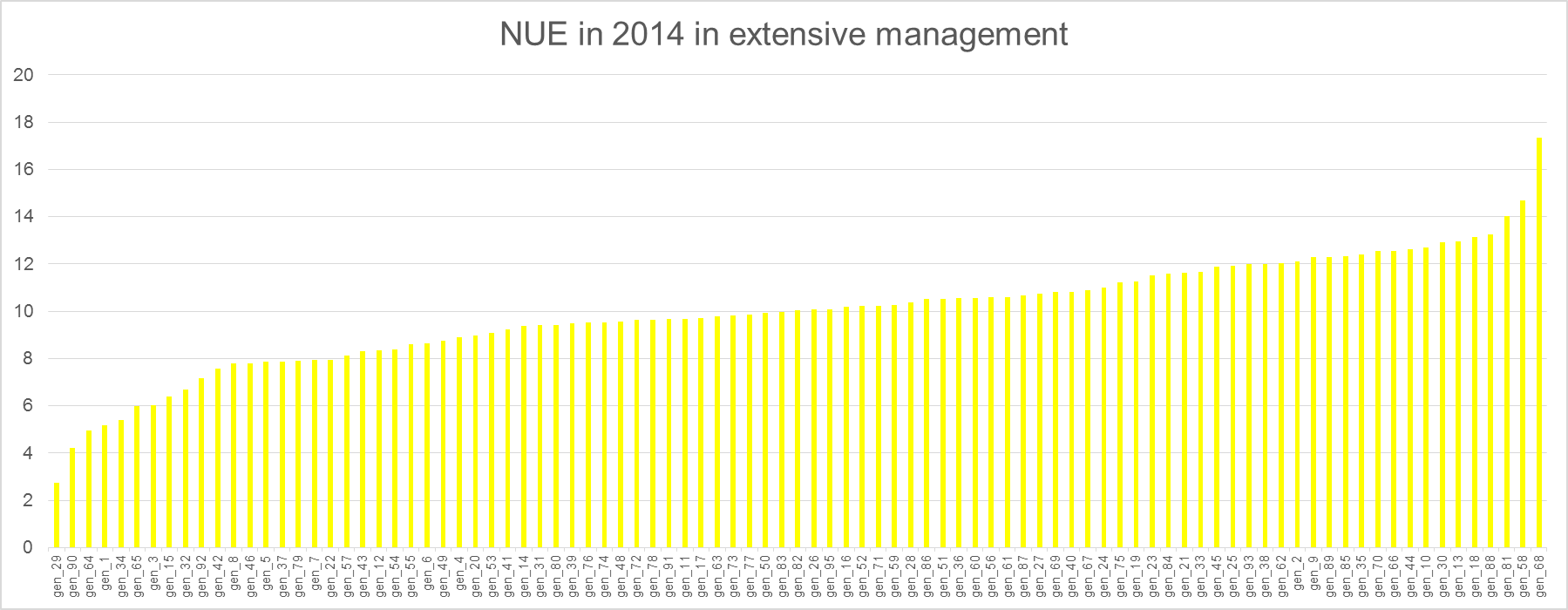


**S4 Fig. Graphs showing the phenotypic distribution of NUE in 2014 in intensive management.**


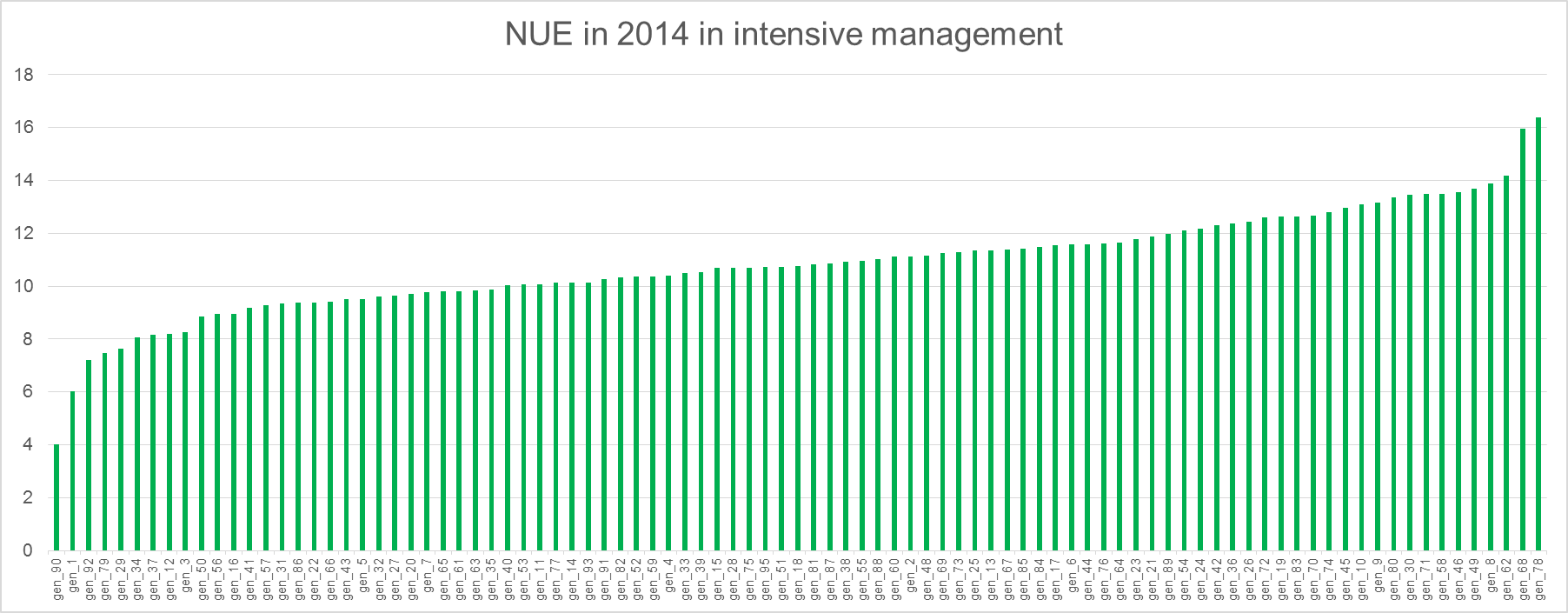


**S4 Fig. Graphs showing the phenotypic distribution of NUE in 2015 in extensive management.**


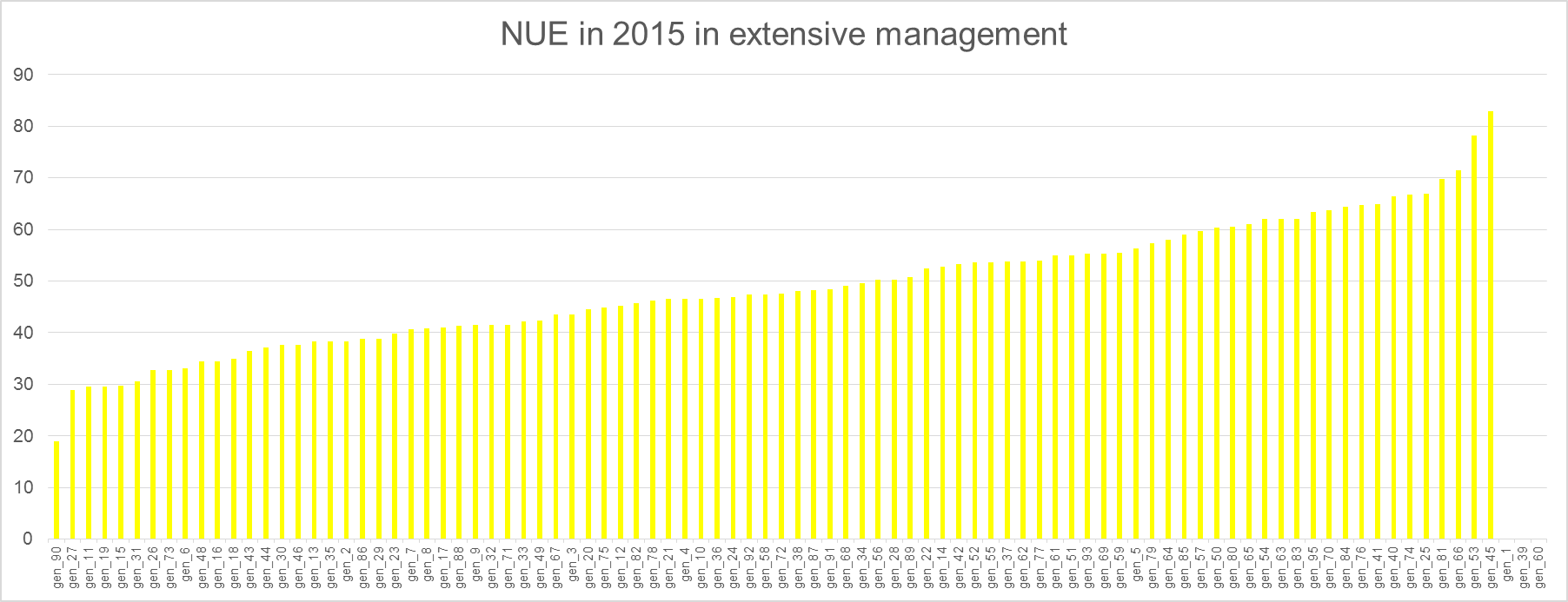


**S4 Fig. Graphs showing the phenotypic distribution of NUE in 2015 in intensive management.**


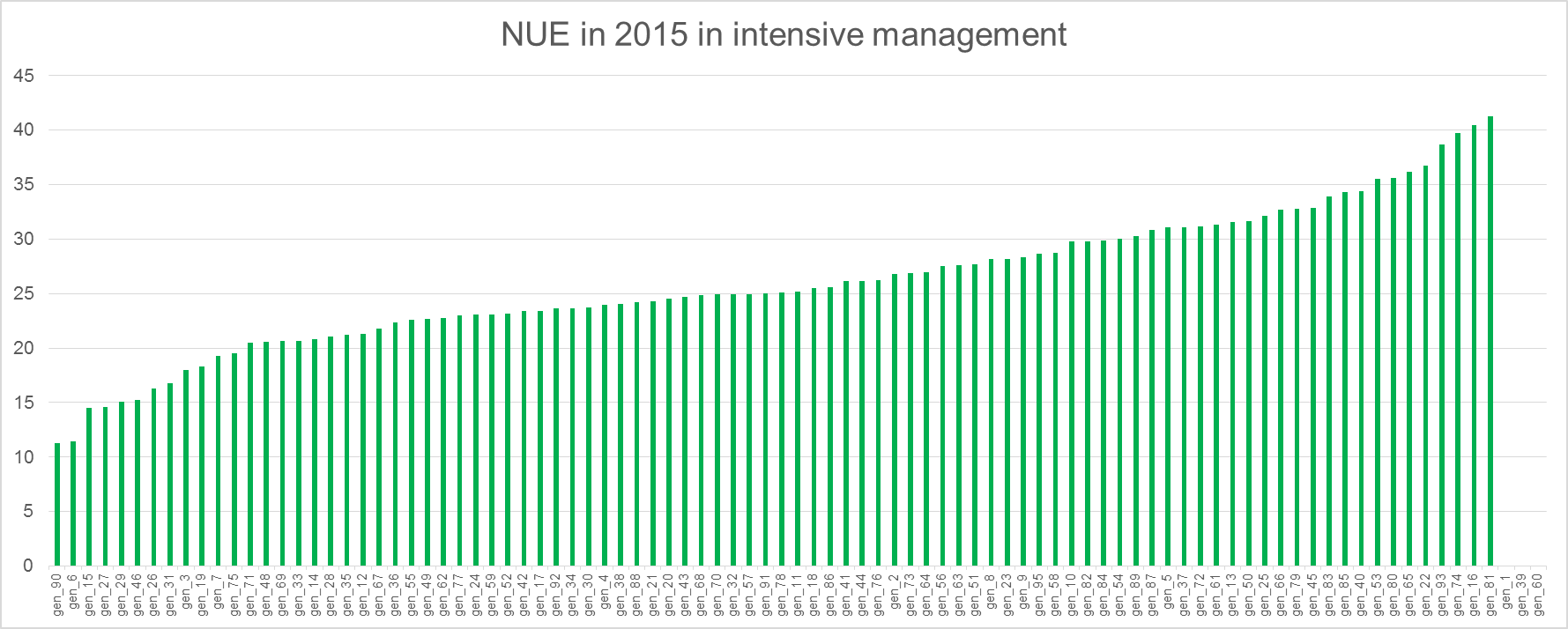

Supplement: S4 Fig — (DOCX) [file pone.0189265.s009.docx]
